# NirA is a Cyanide‐Tolerant Nitrite Reductase Which Protects *Pseudomonas aeruginosa* From Self‐Poisoning

**DOI:** 10.1111/1758-2229.70256

**Published:** 2025-12-09

**Authors:** Samuel Fenn, Carey Lambert, Dimitra Panagiotopulou, Ruth Massey, Miguel Cámara

**Affiliations:** ^1^ National Biofilms Innovation Centre, Biodiscovery Institute, School of Life Sciences University of Nottingham Nottingham UK; ^2^ Biosciences Institute, School of Microbiology University College Cork Cork Ireland; ^3^ APC Microbiome Ireland University College Cork Cork Ireland

## Abstract

Host production of nitric oxide in response to *P. aeruginosa* results in accumulation of nitrite and nitrate at the infection site, with both utilised for anaerobic respiration to support survival. Nitric oxide and nitrite also act as aerobic respiratory inhibitors. *P. aeruginosa* must overcome these toxic metabolites alongside self‐produced cyanide to persist at the infection site. We previously identified a novel nitrite reductase (NirA) that supports *P. aeruginosa* virulence in a wide range of infection models. In this work, we demonstrate that mutation of *nirA* inhibits growth of *P. aeruginosa* at reduced oxygen tensions in the presence of nitrite or nitrate, with this phenotype shown to be dependent on cyanide. NirA is a siroheme‐dependent enzyme, a classical target for inhibition with cyanide. Biochemical characterisation confirms that NirA is a novel cyanide‐tolerant nitrite reductase, which supports reduction of nitrite in the presence of cyanide. We hypothesise that NirA enables detoxification of nitrite to prevent build‐up of multiple respiratory inhibitors and facilitate cyanide‐resistant aerobic respiration at low oxygen tensions. Through targeting effectors of these resistance mechanisms, we could promote *P. aeruginosa* self‐poisoning and prevent adaptation to the reduced oxygen environment typically encountered by *P. aeruginosa* in biofilms and during infection.

## Graphical abstract

Infection‐mediated oxygen limitation triggers *P. aeruginosa* cyanide production. Cyanide inhibits heme‐dependent enzymes, including NirB and NirS of the assimilatory and dissimilatory nitrate reduction pathways. The presence of cyanide upregulates *nirA*, which facilitates cyanide‐tolerant nitrite reduction, detoxifying the respiratory inhibitor nitrite. This supports the growth of *P. aeruginosa* under cyanogenic conditions. Created in BioRender. Fenn, S. (2025), https://BioRender.com/ex2drlv.

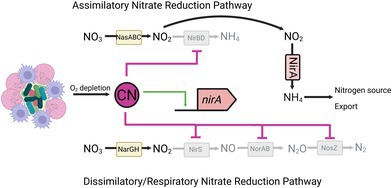

## 1 Introduction

*Pseudomonas aeruginosa* is a genetically versatile opportunistic pathogen capable of colonising diverse environments and hosts (Gellatly and Hancock 2013). This ability to colonise these hosts is underpinned by the utilisation of multiple metabolic pathways, facilitating survival and persistence in host tissues during infection (Gellatly and Hancock 2013; La Rosa et al. 2019; Turner et al. 2014). As a member of the multi‐drug resistant ESKAPE pathogens ( *Enterococcus faecium* , *Staphylococcus aureus* , *Klebsiella pneumoniae* , *Acinetobacter baumannii* , *Pseudomonas aeruginosa* and *Enterobacter* sp.), there is an urgent need for the development of new antimicrobials to combat infections caused by these organisms. Understanding the fundamental metabolic processes required for colonisation and subsequent disease manifestation could pave the way for novel antimicrobial development (Boucher et al. 2009; Miller and Arias 2024).

*P. aeruginosa* possesses a plethora of virulence factors which enable this pathogen to infect multiple sites such as the respiratory tract, soft tissue, burn wounds, urinary tract, bloodstream and cornea. Each of these infection sites is a unique environment, requiring alterations in metabolism and virulence factor profiles to ensure survival and continuation of disease (Gellatly and Hancock 2013; La Rosa et al. 2019; Turner et al. 2014). Availability of carbon, nitrogen and oxygen varies at infection sites and influences the outcome of disease. The two‐component regulatory systems CbrAB and NtrBC form a network which regulates the carbon/nitrogen balance in *P. aeruginosa* , with both systems being essential for full virulence of *P. aeruginosa* (Li and Lu 2007; Yeung et al. 2014; Alford et al. 2020; Alford et al. 2021). Mutation of *cbrA* increased macrophage phagocytosis and reduced virulence in an acute murine lung and peritoneal infection model (Yeung et al. 2014). Loss of *ntrBC* phenocopies mutation of *cbrA*, with increased phagocytosis of mutated strains leading to reduced virulence in an acute murine lung infection model (Alford et al. 2020; Alford et al. 2021).

Macrophages and polymorphonuclear leukocytes (PMNs) influence infection‐specific metabolism through the reduction of oxygen availability and the production of nitric oxide. Once *P. aeruginosa* is detected, PMNs and macrophages migrate to the site of infection and utilise locally dissolved oxygen to support the production of antimicrobial superoxide and nitric oxide to combat invading organisms through the respiratory burst (Kolpen et al. 2010; Kolpen et al. 2014). The loss of oxygen creates a microaerobic/hypoxic environment, inhibiting aerobic respiration, whilst nitric oxide is ultimately oxidised to nitrite or nitrate (Kolpen et al. 2010; Kolpen et al. 2014; Line et al. 2014; Scoffield and Wu 2016). Adaptation to the metabolic landscape imparted by the host is key to survival at the host site. *P. aeruginosa* can utilise host‐derived nitrite and nitrate as both a single nitrogen source for biomass synthesis and as a terminal electron acceptor in anaerobic respiration (Sparacino‐Watkins et al. 2014; Romeo et al. 2012). Assimilatory nitrate reduction reduces nitrate to ammonium for use in amino acid synthesis, whilst denitrification reduces nitrate to dinitrogen gas generating proton motive force (Sparacino‐Watkins et al. 2014; Romeo et al. 2012). This allows *P. aeruginosa* to use the host immune response to promote survival during infection with the respiration of nitrate/nitrite previously linked to persistence and virulence (Line et al. 2014; Van Alst et al. 2009; Van Alst et al. 2007).

We previously identified that mutation of a novel ammonium‐forming nitrite reductase NirA (PA4130) attenuated multiple‐virulence factor production and contributed to virulence in phylogenetically diverse infection models (Fenn et al. 2021). NirA has been identified as part of the cyanide‐inducible gene cluster PA4129‐34 and is regulated by the cyanide‐responsive regulator MpaR, which is part of this cluster (Frangipani et al. 2014; Smiley et al. 2024). Hydrogen cyanide is a potent respiratory inhibitor maximally produced by *P. aeruginosa* at high cell densities and reduced oxygen concentration in a quorum‐sensing and ANR‐dependent manner (Pessi and Haas 2000). With *P. aeruginosa* encoding another ammonium‐forming nitrite reductase (NirBD) and a nitric‐oxide‐forming reductase (NirS) (Romeo et al. 2012; Van Alst et al. 2009), we sought to distinguish the exact function of NirA to unravel how it contributes to *P. aeruginosa* virulence.

In this study, we determine that NirA is not responsive to nitrogen‐source availability and does not encode a classical assimilatory nitrite reductase. We demonstrate that mutation of *nirA* compromises growth of *P. aeruginosa* in the presence of nitrite/nitrate in an oxygen‐dependent manner. This inhibition was shown to be dependent on cyanide, with growth and enzymatic assays revealing that NirA is a cyanide‐resistant nitrite reductase capable of reducing nitrite at cyanide concentrations above what is detected in nature. This prevents dual poisoning with the two respiratory inhibitors, nitrite and cyanide, a condition *P. aeruginosa* often encounters during infection.

## 2 Results

### 2.1 NirA Is Not an Assimilatory Nitrite Reductase

Our previous work demonstrated that *nirA* encodes an ammonium‐forming nitrite reductase (Fenn et al. 2021). NirA is required for *P. aeruginosa* virulence in multiple disease models, although the mechanistic role it plays is still unclear (Fenn et al. 2021). In both plants and bacteria, NirA homologues are part of the assimilatory nitrate reduction pathway, producing ammonium as a nitrogen source for biomolecule synthesis (Sparacino‐Watkins et al. 2014). The prototypical assimilatory nitrite reductase of *P. aeruginosa* has previously been shown to be *nirBD*, catalysing production of ammonium during N source limitation (Romeo et al. 2012).

**FIGURE 1:**
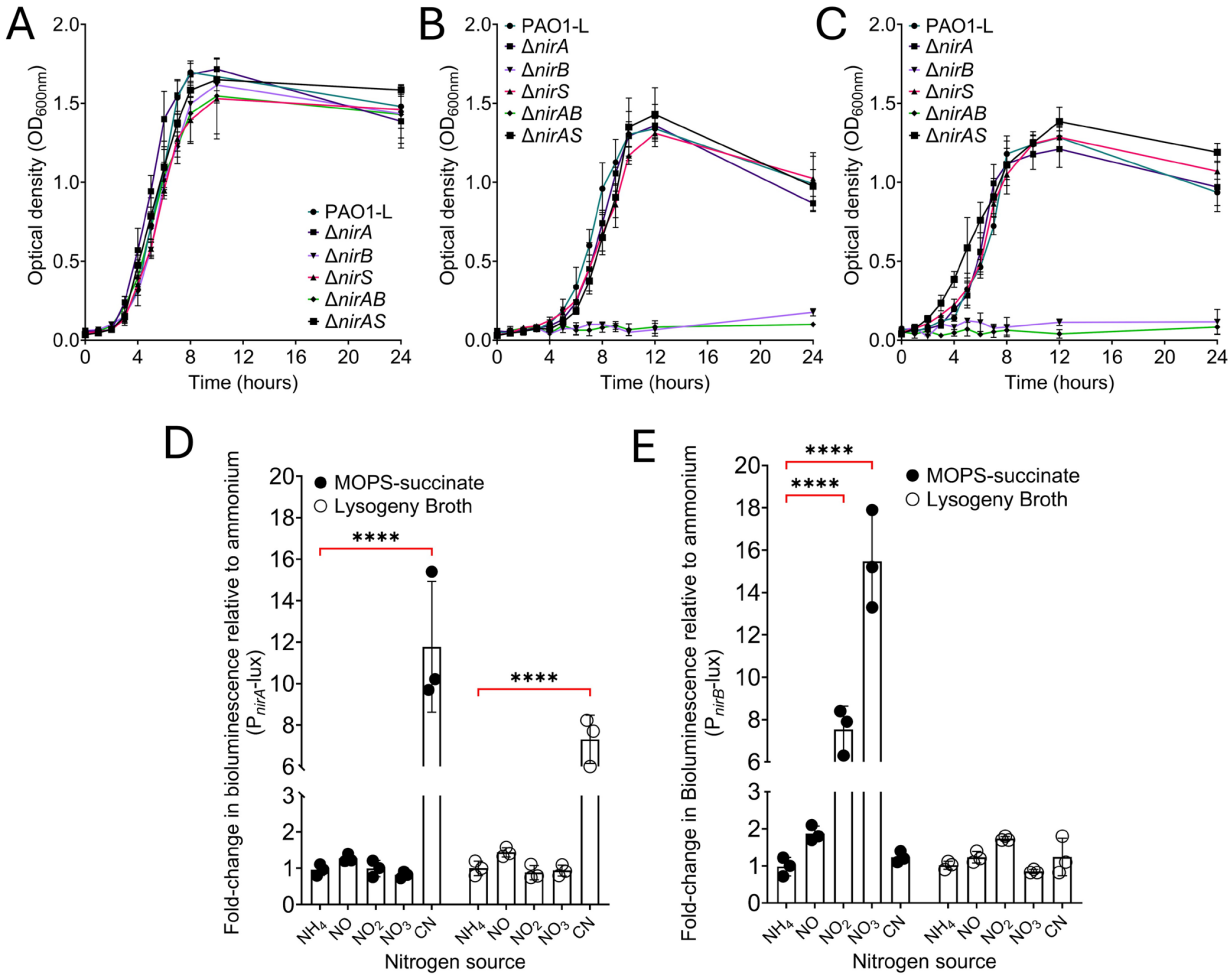
NirA does not function as an assimilatory nitrite reductase under aerobic culture conditions. Aerobic culture of nitrite reductase mutants on MOPS‐succinate minimal media with ammonium (A), nitrite (B) and nitrate (C) as single nitrogen sources. Deletion of *nirA* or *nirS* did not compromise growth of PAO1‐L when cultured on ammonium, nitrite and nitrate under aerobic conditions as both single and double mutants. Loss of *nirB* prevented the use of nitrite or nitrate as a single nitrogen source, consistent with its role as an assimilatory nitrite reductase. Response of P_*nirA*_ (D) and P_*nirB*_‐lux (E) transcriptional reporters to the presence of nitrogen sources. Transcriptional activity of *nirA* was only induced in the presence of cyanide, with no response exhibited by the presence of nitrogen sources. Bioluminescence driven by P_*nirB*_ was activated by single nitrogen sources in minimal media only. Data were collated from three independent experiments, with error bars representing standard deviation. Each experiment consists of three technical replicates. Significance determined as ****p < 0.0001 using Dunnett's multiple comparisons test.

Both NirA and NirBD perform the same molecular function, catalysing the 6‐electron reduction of nitrite to ammonium, whilst NirS reduces nitrite to nitric oxide (Romeo et al. 2012; Fenn et al. 2021). To determine if there is functional redundancy between NirA, NirBD and NirS, *nirB and nirS* mutants were made in PAO1‐L, with *nirAB* and *nirAS* double mutants also constructed using PAO1‐L ∆*nirA*. Single and double deletion of both *nirA* and *nirS* did not impact replication of *P. aeruginosa* when cultured aerobically in MOPS‐succinate minimal media with ammonium, nitrite and nitrate as single nitrogen sources (Figure 1). Mutants harbouring a *nirB* deletion failed to grow on nitrite and nitrate as single N sources (Figure 1B,C), supporting the previous findings that *nirBD* is the primary assimilatory nitrite reductase of *P. aeruginosa* (Romeo et al. 2012). No significant growth defect was exhibited for any strain cultured aerobically in LB supplemented with ammonium, nitrite, or nitrate (Figure S1).

Categorisation of NirA as an assimilatory nitrite reductase is dependent on the ability to respond to low N source availability and exploit alternatives. *P. aeruginosa nirBD* transcription is only activated under conditions when preferred nitrogen sources (amino acids and ammonium) are less abundant, whilst nitrate/nitrite availability is high (Romeo et al. 2012). Regulation of *nirA* has been shown to be indirectly dependent on QS through endogenous cyanide production (Frangipani et al. 2014) however, the effect of nitrogen sources on the expression of this gene has yet to be assessed. To determine if *nirA* transcription is responsive to N sources, P_*nirA*_‐lux and P_*nirB*_‐lux transcriptional reporters were constructed in pmini‐CTX‐lux‐Gm and conjugated into PAO1‐L. Exposure of P_*nirA*_‐lux to ammonium, nitric oxide, nitrite and nitrate in both minimal and complex media resulted in no significant increase in P_*nirA*_ driven bioluminescence, with only hydrogen cyanide yielding an increase in P_*nirA*_ activity (Figure 1D). Meanwhile, bioluminescence driven by P_*nirB*_ only increased in minimal media with nitrate and nitrite (Figure 1E). The lack of P_*nirA*_ response to nitrogen sources combined with the absence of a growth defect when cultured on nitrite/nitrate indicates that NirA is not a classical assimilatory nitrite reductase.

### 2.2 NirA Is Required for Growth Under Reduced Oxygenation When Exposed to Nitrite and Nitrate

Upregulation of *nirA* by HCN indicates the role of this enzyme in vivo is likely to be under reduced oxygenation (Frangipani et al. 2014; Pessi and Haas 2000). During infection, *P. aeruginosa* encounters heterogenous oxygen concentrations and elevated levels of nitrite/nitrate through biofilm formation and the actions of polymorphonuclear leukocytes (Kolpen et al. 2010; Kolpen et al. 2014; Van Alst et al. 2007). This provides optimal conditions for upregulation of *P. aeruginosa* HCN production. To investigate the impact of oxygenation on the growth of PAO1‐L ∆*nirA*, we measured both biofilm formation and growth kinetics under reduced oxygen conditions.

**FIGURE 2:**
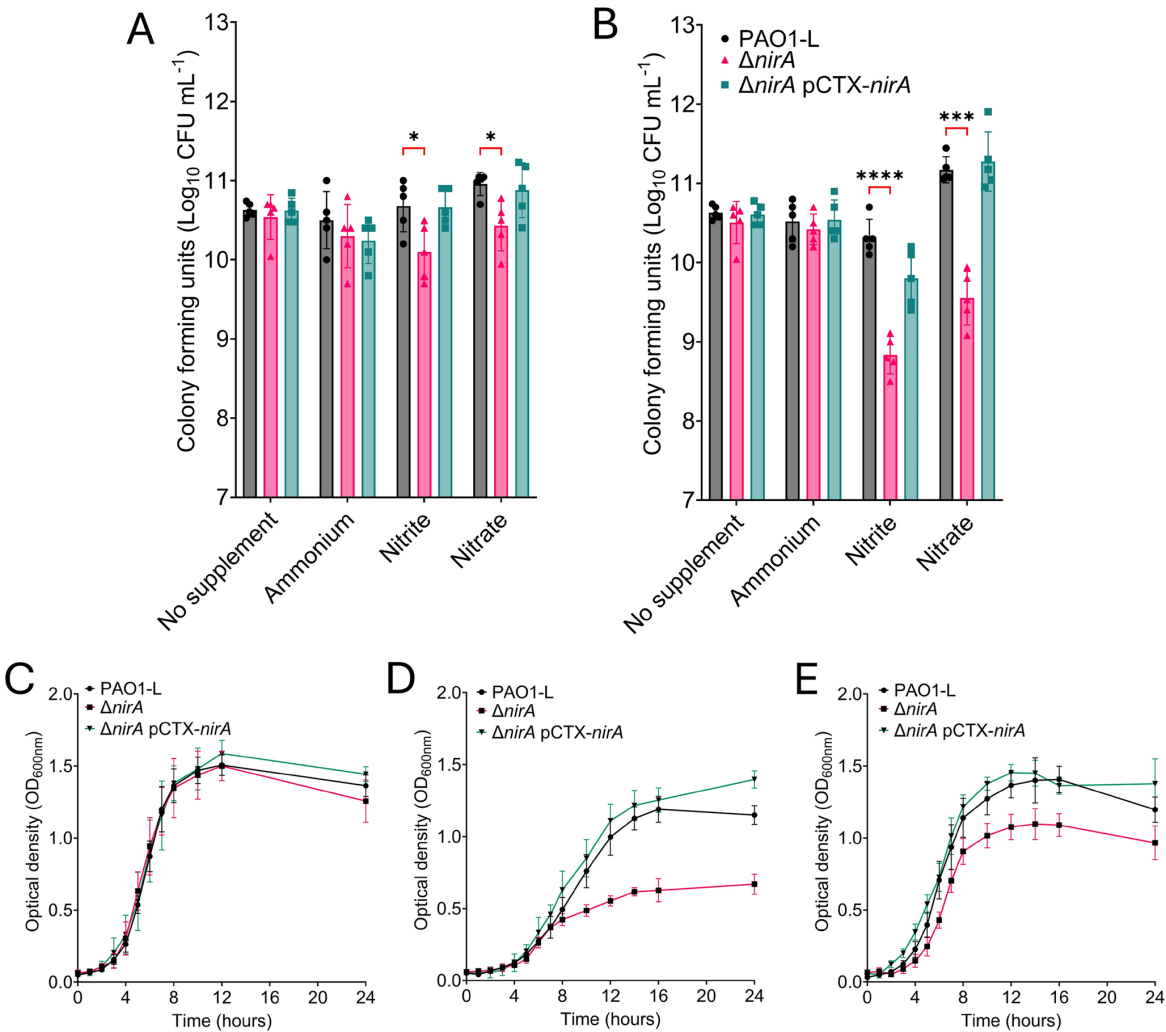
NirA‐dependent reduction of nitrite occurs in an oxygen‐dependent manner. Colony biofilms cultured on LB with ammonium, nitrite or nitrate established under aerobic (A) and microaerobic (2% O_2_) (B) conditions for 72 h. Deletion of *nirA* reduced colony‐forming unit recovery when cultured in the presence of nitrite and nitrate, with reduced oxygenations enhancing this phenotype. Growth kinetics of ∆*nirA* and associated complement when cultured on MOPS‐succinate with ammonium (C), nitrite (D) and nitrate (E) under microaerobic conditions. Loss of *nirA* compromised growth in MOPS‐succinate‐nitrite/nitrate, with genetic complementation restoring wild‐type growth kinetics. For colony biofilms, data were collated from five individual experiments with at least three technical replicates. For growth kinetics, data were collated from three experiments with error bars representing standard deviation. Significance determined at *p < 0.05, ***p < 0.001 and ****p < 0.0001 using Dunnett's multiple comparisons test.

Colony biofilms were established on LB supplemented with ammonium, nitrite and nitrate using polycarbonate discs to facilitate accurate colony‐forming unit (CFU) determination. Under aerobic conditions, a modest 0.5 log decrease in CFU recovery was exhibited by the *nirA* mutant in the presence of nitrite and nitrate (Figure 2A). The impact of nitrite and nitrate was magnified when these biofilms were established at 2% O_2_, with a 1.7 and 1.5 log reduction in CFU observed for PAO1‐L ∆*nirA* (Figure 2B). Under both aerobic and microaerobic conditions, genetic complementation restored the ∆*nirA* phenotype. This pattern was also observed in artificial sputum media (ASM), with the presence of nitrite and nitrate reducing CFU recovery of a *nirA* mutant in an oxygen‐dependent manner (Figure S2). Growth kinetics were tracked in MOPS‐succinate media with varying nitrogen sources at 2% O_2_. When cultured on ammonium as an N source, ∆*nirA* growth kinetics mimic the wild type (Figure 2C). However, when exposed to nitrite or nitrate at low oxygen tensions, a *nirA*‐null mutant demonstrated a cessation in growth (Figure 2D,E). This impact was most prominent when cultured on nitrite or nitrate as a single nitrogen source; however, a significant decrease in OD_600nm_ was also exhibited when LB and ASM were supplemented with 5 mM nitrite (Figure S3). On complex media, the addition of nitrate did result in *nirA* growth attenuation (Figure S3). The exacerbated growth defect induced by culture under reduced oxygenation supports the hypothesis that the nitrite reduction function of *nirA* is important under low oxygen tensions. Infection microenvironments are often depleted in oxygen, and biofilms naturally form oxygen gradients, meaning the ability to persist at an infection site is dependent on *P. aeruginosa* surviving under these conditions.

### 2.3 NirA Is a Cyanide‐Tolerant Nitrite Reductase

When cultured on MOPS‐succinate‐nitrite/nitrate under reduced oxygenations, PAO1‐L ∆*nirA* initially replicated before growth cessation. Given that a *nirA* mutant possesses intact *nirBD*, this strain should be able to replicate on nitrite or nitrate as a single nitrogen source. However, the growth cessation observed indicates that *nirB* activity is being inhibited at reduced oxygenations. Reduced oxygenation induces hydrogen cyanide synthesis, with *nirA* identified to be part of a gene cluster induced by cyanogenesis via the cyanide‐responsive regulator MpaR (Frangipani et al. 2014; Smiley et al. 2024). Cyanide binds to ferric heme iron, blocking the transport of electrons (Sugishima et al. 2007). Whilst best described as a respiratory chain inhibitor through binding of heme in cytochrome C and cytochrome C oxidases, it is also known to interfere with heme cofactors of other enzymes, such as the heme _*cd*1_ of nitric‐oxide forming nitrite reductase (Sun et al. 2002), and siroheme of ammonium forming nitrite reductases such as NirA homologues and NirBD (Coleman et al. 1978; Kang et al. 1987; Kaufman et al. 1993). We hypothesised that this inhibitor of NirB function was cyanide, as this toxic secondary metabolite is produced maximally under reduced oxygenation. Whilst NirA and NirBD are structurally unrelated, both enzymes are dependent on the prosthetic group siroheme to mediate electron transfer. This makes upregulation of *nirA* by cyanide counterintuitive since this enzyme is upregulated by an inhibitor. This gene is part of the cyanide‐inducible gene cluster regulated by MpaR and led to the hypothesis that NirA is a cyanide‐resistant nitrite reductase.

**FIGURE 3:**
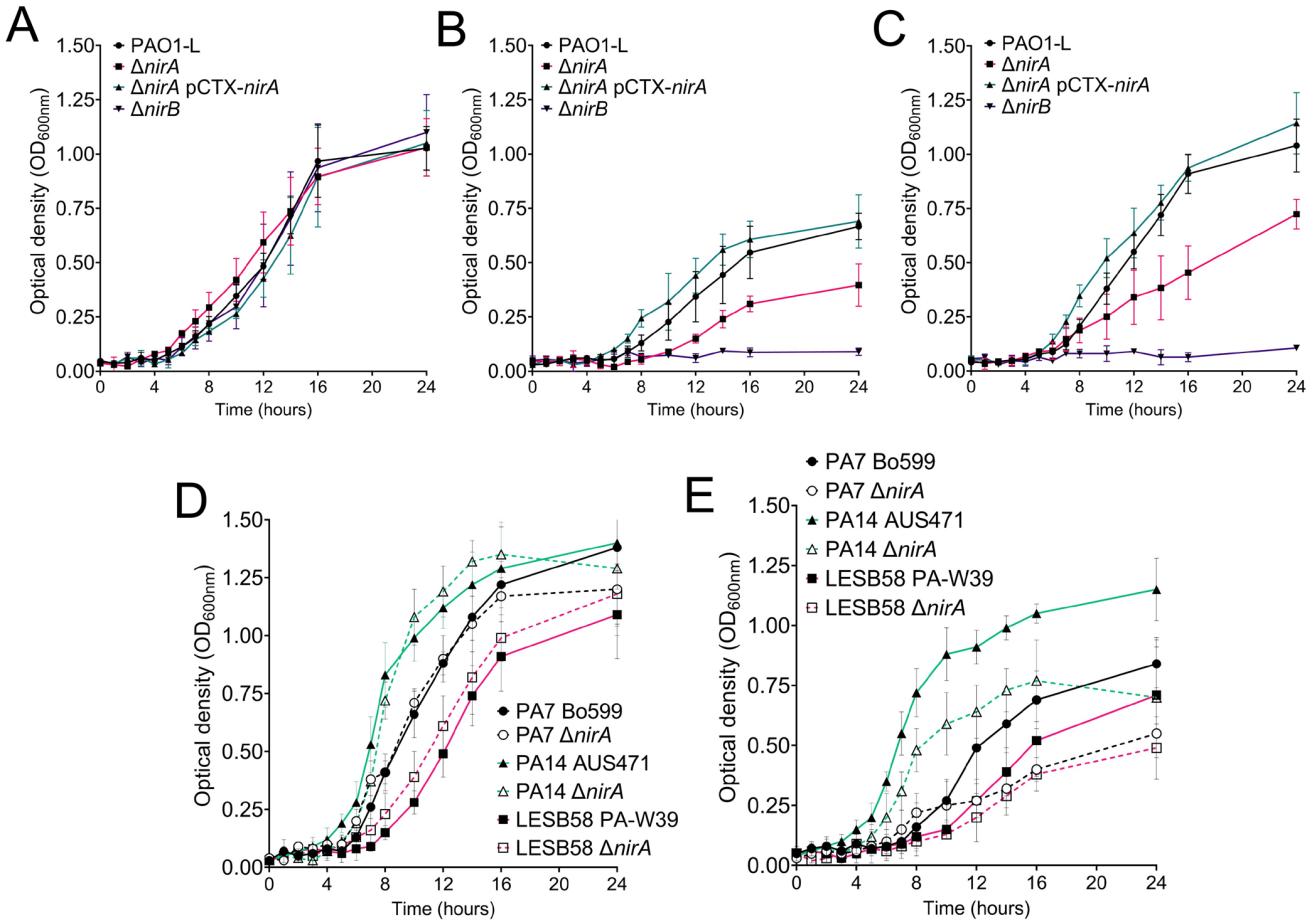
Loss of NirA compromises growth on nitrite and nitrate in a cyanide‐dependent manner. Aerobic culture of PAO1‐L nitrite reductase mutants on MOPS‐succinate minimal media supplemented with 50 μM cyanide using ammonium (A), nitrite (B) and nitrate (C) as single nitrogen sources. Addition of cyanide resulted in an extended lag phase for all cultures. Growth on ammonium was unimpeded by mutation of *nirA* and *nirB*, with all strains resembling the isogenic wild‐type strain. When using nitrite or nitrate as a single nitrogen source, PAO1‐L ∆*nirA* is impacted in the presence of KCN, phenocopying the growth defect previously exhibited by a *nirA* mutant under reduced oxygenation. Supplementation of a PAO1‐L ∆*nirB* with KCN does not enable NirA to functionally substitute as an assimilatory nitrite reductase in minimal media. Aerobic culture of clinical *P. aeruginosa* strains deleted for *nirA* on MOPS‐succinate minimal media supplemented with 100 μM cyanide using ammonium (D) and nitrite (E) as nitrogen sources. When *nirA* mutants are cultured on ammonium in the presence of cyanide, growth kinetics are similar to the isogenic wild‐type strains. Clinical *nirA* mutant growth kinetics are compromised when cultured on nitrite in the presence of cyanide, although the magnitude of this impact is strain specific. Data were collated from a minimum of three independent experiments with error bars representing standard deviation.

To confirm that cyanide‐based inhibition of NirB is responsible for the growth defect exhibited by PAO1‐L ∆*nirA* under reduced oxygen tensions, we monitored growth kinetics in the presence of 100 μM cyanide under aerobic conditions in MOPS‐ succinate minimal media. In ∆*nirA* strains, NirBD is the only active ammonium‐forming reductase; therefore, utilisation of nitrite or nitrate as a single nitrogen source is solely dependent on NirBD. Previously, no growth defect was exhibited by a *nirA*‐null strain when cultured on nitrite/nitrate (Figure 1B,C). When cultured in the presence of cyanide, growth kinetics in the presence of ammonium of all strains were comparable to the wild type (Figure 3A). However, mutation of *nirA* impeded replication of PAO1‐L in the presence of nitrite and nitrate (Figure 3B,C), phenocopying the defect exhibited during culture under reduced oxygenations (Figure 2D,E). These assays were repeated in the presence and absence of cyanide using *nirA* mutants constructed in clinical *P. aeruginosa* strains PA7 Bo599, PA14 AUS471 and LESB58 PAW‐39 (Fenn et al. 2021; Freschi et al. 2015). The *P. aeruginosa* clinical strains demonstrated a similar phenotype, with cyanide supplementation impacting growth kinetics when using nitrite as a single nitrogen source, although the magnitude of this effect varied between strains (Figure 3D,E). In LB, a *nirA*‐dependent growth defect is only observed when cultured in the presence of nitrite under reduced oxygen tension. Addition of cyanide to aerobic LB cultures supplemented with nitrite recapitulates this phenotype, confirming NirA plays a role in detoxifying nitrite in the presence of cyanide as opposed to being employed as an assimilatory reductase (Figure S4).

**FIGURE 4:**
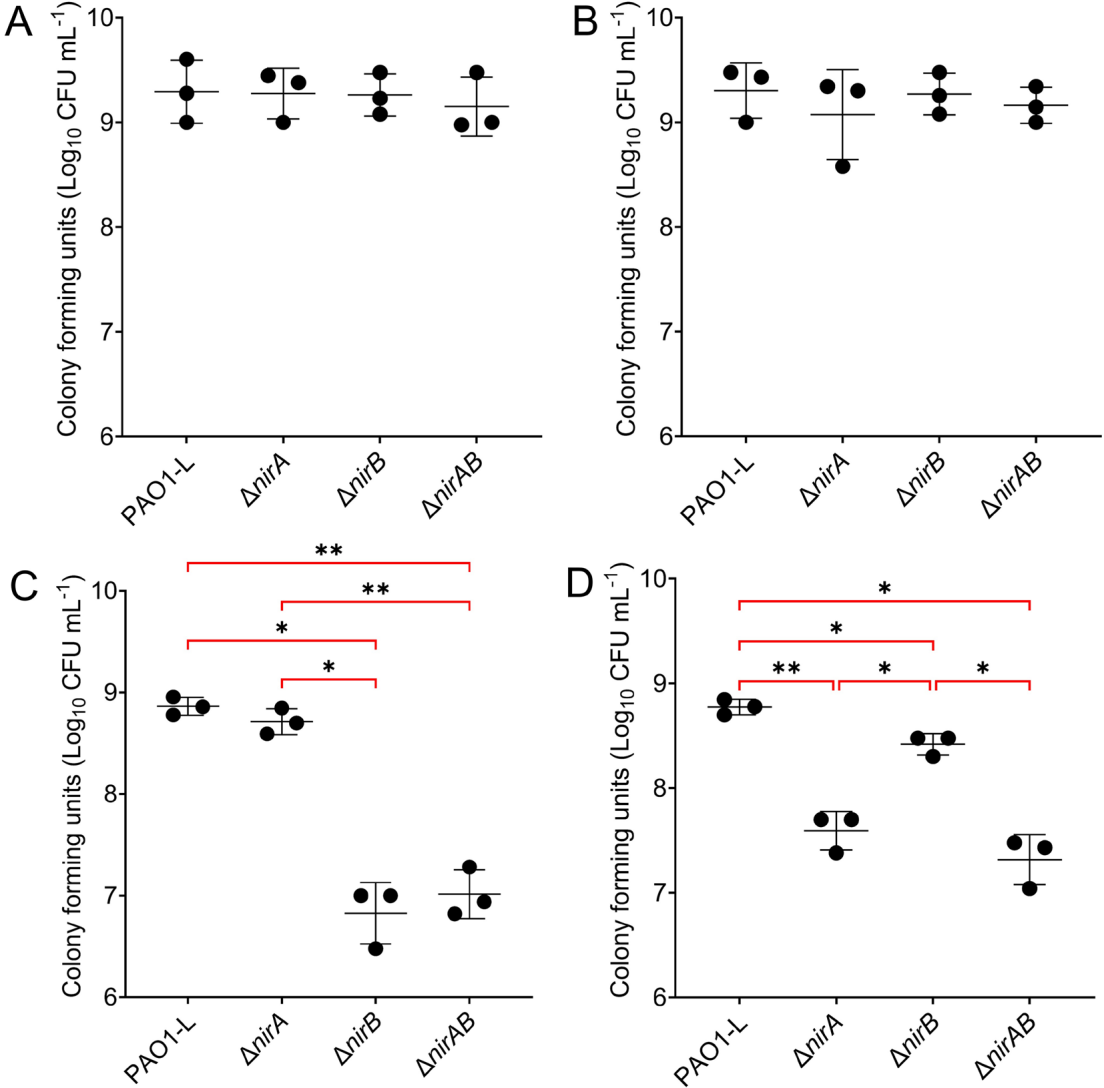
NirA functionally substitutes for NirB at high‐cell density in the presence of cyanide. PAO1‐L nitrite reductase mutant cultured overnight in MOPS‐succinate‐NH_4_ sub‐cultured at high cell density (1 × 10^8^) into MOPS‐succinate‐NH_4_ (A), NH_4_ + KCN (B), NO_2_ (C) and NO_2_ + KCN (D). Cyanide supplemented at 50 μM where applicable. Mutation of nitrite reductase mutants did not impair PAO1‐L use of ammonium as a single nitrogen source in the absence/presence of cyanide (A, B). When transferred to MOPS‐succinate‐nitrite, strains without a functional NirB failed to replicate and succumbed to nitrite toxicity whilst loss of NirA demonstrated no significant effect (C). In comparison, when transferred to MOPS‐succinate‐NO2 + KCN a single *nirB* deletion mutant could replicate, whilst a *nirA* mutant was unable to grow (D). Loss of both *nirA* and *nirB* inhibited growth on nitrite+KCN indicating that NirA functionally substitutes for NirB at high cell density in the presence of cyanide. Data displayed represent CFU recovery after 24‐H static incubation at 37°C following transfer to the described condition. Tracking of CFU recovery over time is available in Figure S5. Each point represents an independent experiment with at least three replicates per experiment. Data were collated with lines representing the mean of the three independent experiments and error bars the standard deviation. Significance determined at *p < 0.05, **p < 0.01, using Dunnett's T3 multiple comparisons test.

Unexpectedly, the addition of cyanide to PAO1‐L ∆*nirB* did not restore growth on nitrite or nitrate despite the addition of cyanide inducing *nirA* (Figure 3B,C). We hypothesised that this is due to the low initial inoculum used to establish these growth curves. NirA requires the presence of other proteins such as ferredoxins to mediate nitrite reduction. Given the role of quorum sensing in the regulation of cyanide production, we hypothesised that NirA would only be functional at higher cell densities. To test this, we first cultured nitrite reductase mutant strains in MOPS‐succinate‐NH_4_, pelleted and washed the cells, and reinoculated strains at 1 × 10^8^ in MOPS‐succinate‐NH_4_, NH_4_ + KCN, NO_2_ and NO_2_ + KCN. We then tracked CFU recovery over 24 h in this media to determine if NirA can functionally substitute for NirB at higher cell densities. As expected, no difference in CFU recovery was observed between nitrite reductase mutants transferred into NH_4_ and NH_4_ + KCN (Figures 4A,B and S5A,B). When nitrite reductase mutants were transferred to MOPS‐succinate‐NO_2_, the loss of *nirA* did not impact utilisation of nitrite, whilst the mutation of *nirB* compromised the ability of *P. aeruginosa* to use nitrite as a single nitrogen source for biomass production (Figures 4C and S5C). However, when transferred to MOPS‐succinate‐NO_2_ + KCN, a *nirB* mutant could utilise nitrite for growth (Figures 4D and S5D). Double mutation of *nirA* and *nirB* resulted in the loss of the ability to utilise nitrite in the presence of cyanide, demonstrating that NirA can functionally substitute for NirB under these defined environmental conditions (Figures 4D and S5D). This highlights the importance of both cell density and the presence of cyanide for NirA function.

**FIGURE 5:**
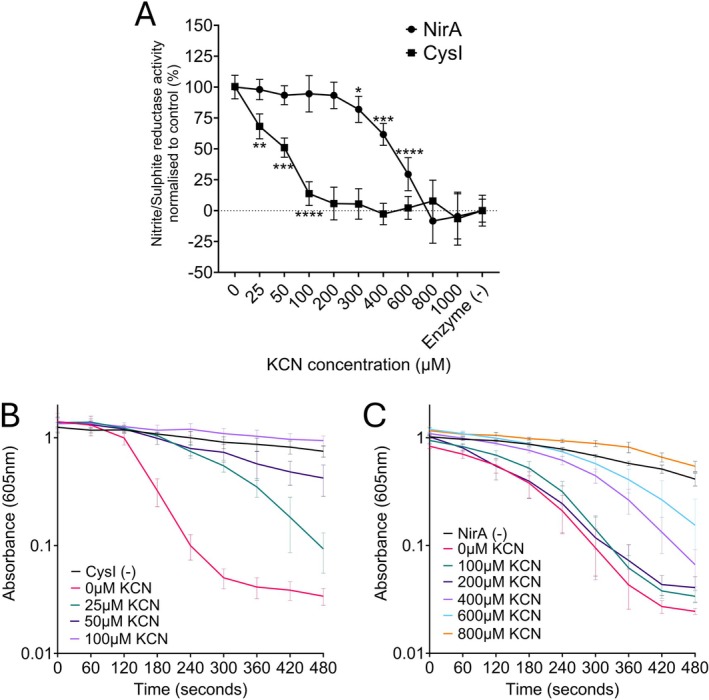
NirA catalyses nitrite reduction in the presence of cyanide. Comparison of NirA and CysI activity in the presence of increasing cyanide concentrations (A). End‐point methyl‐viologen oxidation assays were performed for 10 min under anaerobic conditions using nitrite and sulphite as electron acceptors for NirA and CysI, respectively. Percentage activity and significance were denoted through comparison to the control assay containing no KCN. CysI activity was significantly inhibited by the presence of cyanide even at the lowest concentrations tested. NirA activity was unaffected in the presence of 200 μM cyanide, with significant activity detected at 400–600 μM. Kinetic methyl‐viologen assays were performed in the presence of KCN using CysI (B) and NirA (C). Drastic reduction in CysI activity occurred, with activity severely impeded by the presence of 25–50 μM KCN, and complete inhibition at 100 μM. In comparison, methyl‐viologen oxidation mediated by NirA at 200 μM KCN was kinetically similar to the 0 μM KCN control. Subsequent increases in KCN inhibited NirA methyl‐viologen oxidation; however, significant activity was still detected at 600 μM KCN, with complete inhibition occurring at 800–1000 μM. Assays were performed using three preparations of CysI and NirA, with data were collated from three independent experiments. Data were collected from Tecan Infinite with a gas control module to maintain anaerobic conditions. Significance was determined as *p < 0.05, **p < 0.01, ***p < 0.001 and ****p < 0.0001 using Dunnett's multiple comparisons test.

We next sought to confirm NirA cyanide‐resistance biochemically through comparison to NirB activity; however, despite numerous attempts, we were unable to obtain soluble NirB. Instead, PAO1‐L CysI was selected as a control enzyme for the assessment of cyanide sensitivity. CysI possesses 61% amino acid sequence similarity to NirA; however, it uses sulphite instead of nitrite as an electron acceptor, catalysing the 6‐electron reduction of sulphite to hydrogen sulphide. Enzymes of this family reduce both nitrite/sulphite, although they demonstrate a preference for an electron acceptor, which informs nomenclature (Kaufman et al. 1993). KCN inhibition was measured as a dose–response curve using a methyl‐viologen oxidation assay, as previously performed (Fenn et al. 2021). Enzymes were pre‐incubated with KCN for 5 min prior to the addition of nitrite or sulphite to ensure cyanide binding in the absence of a competitor. The activity of CysI in the presence of KCN was impacted at the lowest concentrations of 25 μM, with complete loss of activity at 100 μM (Figure 5A). Meanwhile, NirA‐mediated methyl viologen oxidation was unimpeded at 200 μM KCN, with significant activity detected at 600 μM in end‐point assays (Figure 5A). In kinetic assays, CysI mediated reduction was severely impacted at the lowest cyanide concentration tested, whilst NirA was unaffected by the addition of 200 μM cyanide (Figure 5B,C). At concentrations above this, an extended lag in NirA enzyme activity was exhibited, with activity completely abolished at 800 μM (Figure 5C). Taken together with the cyanide‐dependent growth inhibition, these assays confirm that NirA is a cyanide tolerant nitrite reductase.

## 3 Discussion

In a previous study, we identified *nirA* as an ammonium‐forming ferredoxin‐dependent nitrite reductase which contributes to *P. aeruginosa* virulence in multiple infection models (Fenn et al. 2021). Whilst the biochemical role of NirA was understood, the biological impact of this enzyme remained to be elucidated. The ability of *P. aeruginosa* to utilise diverse nitrogen sources is essential for its survival in multiple environments. Nitrate and nitrite can be divided into two main pathways: assimilatory reduction which enables use of single nitrogen sources for amino acid biosynthesis, and dissimilatory reduction which allows use of nitrate and nitrite for respiration in the absence of oxygen (Sparacino‐Watkins et al. 2014; Romeo et al. 2012; Arai 2011). Dissimilatory denitrification produced gaseous dinitrogen; however, pathways describing dissimilatory nitrite reduction to ammonium (DNRA) have been described which enable use of nitrate and nitrite as terminal electron acceptors, whilst simultaneously maintaining ammonium as a usable nitrogen source (Sparacino‐Watkins et al. 2014; Tiso and Schechter 2015).

The prototypical ammonium‐forming nitrite reductase NirBD enables *P. aeruginosa* to use nitrite as a single nitrogen source (Romeo et al. 2012). With NirA also producing ammonium, we investigated functional redundancy in the assimilatory reduction pathway; however, loss of *nirA* does not impact growth kinetics when *P. aeruginosa* is cultured on nitrite or nitrate as a single nitrogen source under aerobic conditions (Figure 1B,C). Comparison of promoter activity between P_*nirA*_ and P_*nirB*_ demonstrates that these enzymes are differentially regulated, with P_*nirB*_ only responding to the presence of nitrite and nitrate as a single nitrogen source, whilst P_*nirA*_ responds in a cyanide‐specific manner (Figure 1A). This led us to conclude that NirA is not an assimilatory nitrite reductase as it is not responsive to nitrogen source availability. DNRA nitrite reductases such as NrfA of *E. coli* also produce ammonium; however, NirA is structurally distinct from this enzyme and is not predicted to be membrane or periplasmically localised (Khlebodarova et al. 2016). This prevents NirA from contributing to energy production and conservation through respiration. The nitric oxide‐forming nitrite reductase NirS contributes to energy production under reduced oxygenation. We confirmed this through culturing under 2% oxygen with a *nirS* mutant demonstrating a slight growth defect similar to *nirA* (Figure S3). Double deletion of *nirAS* results in an additive effect, with loss of both nitrite reductases attenuating growth under reduced oxygenations in the presence of nitrite. This demonstrates that *nirA* and *nirS* play an overlapping role in utilisation and detoxification of nitrite under reduced oxygenation.

Regulation of *nirA* through cyanide was first established by Frangipani and colleagues, with Smiley et al. later demonstrating that this mechanism is dependent on the cyanide‐sensing regulator MpaR (Frangipani et al. 2014; Smiley et al. 2024). Cyanide is an important virulence factor of *P. aeruginosa* , contributing to the killing of host cells and for competition with the host microbiota or other invading bacterial species (Broderick et al. 2008; Nair et al. 2014; Létoffé et al. 2022). Cyanogenesis is maximal at high cell densities under reduced oxygen tension through the dual action of *las*/*rhl* quorum sensing circuit and the oxygen‐sensitive regulator ANR (Pessi and Haas 2000). The growth defect exhibited by ∆*nirA* under reduced oxygenation in both PAO1‐L and clinical strains, combined with the regulatory contribution of cyanide to *nirA* expression, suggested that this enzyme contributed to nitrite reduction in the presence of cyanide in both biofilms and planktonic cultures (Figure 2). Both growth and biochemical assays with purified enzyme confirm that NirA is a novel cyanide‐tolerant nitrite reductase (Figures 3 and 5). NirA activity is unimpeded at 200 μM KCN, which is above physiologically relevant concentrations, with up to 130 μM cyanide detected in the sputum of *P. aeruginosa‐infected* CF patients (Nair et al. 2014).

Supplementation of PAO1‐L ∆*nirB* with cyanide did not restore growth of this strain on nitrite or nitrate despite cyanide inducing transcription of *nirA* (Figure 3B,C). The reason for this is unclear, but this further demonstrates how the functions of NirBD and NirA are not redundant despite catalysing the same chemical reaction. The lack of redundancy could be due to the different electron donors used by NirBD (NADH) and NirA (ferredoxin) (Romeo et al. 2012; Fenn et al. 2021). NirA requires induction of iron–sulphur cluster containing proteins called ferredoxins to mediate electron transport and catalysecatalyse reduction of nitrite to ammonium. Cyanide has not been shown to induce ferredoxin production (Frangipani et al. 2014), thus, whilst NirA will be induced, reduction cannot occur under the conditions tested in this study. With *P. aeruginosa* , cyanide production controlled by quorum‐sensing, we hypothesised that the lack of redundancy between NirA and NirB could be due to the low starting inoculums used for our initial growth curves. By increasing the starting inoculum from 1 × 10^5^ to 1 × 10^8^ we observed that a *nirB* mutant strain could utilise nitrite as a single nitrogen source in the presence of cyanide, with loss of *nirA* inhibiting this growth, indicating NirA can functionally substitute for NirB under these conditions (Figures 4D and S5D). NirA does not fully complement the loss of NirB, with a significant reduction in CFU recovery exhibited between PAO1‐L and a *nirB* mutant with intact *nirA* (Figures 4D and S5D). Combined with the fact that *nirA* and *nirB* are differentially regulated and *nirA* does not respond to N source availability, we believe these enzymes are not redundant and perform distinct roles, with NirB an assimilatory reductase, whilst NirA participates in protecting *P. aeruginosa* from nitrite under conditions where the other nitrite reductases are inactivated or not upregulated.

**FIGURE 6:**
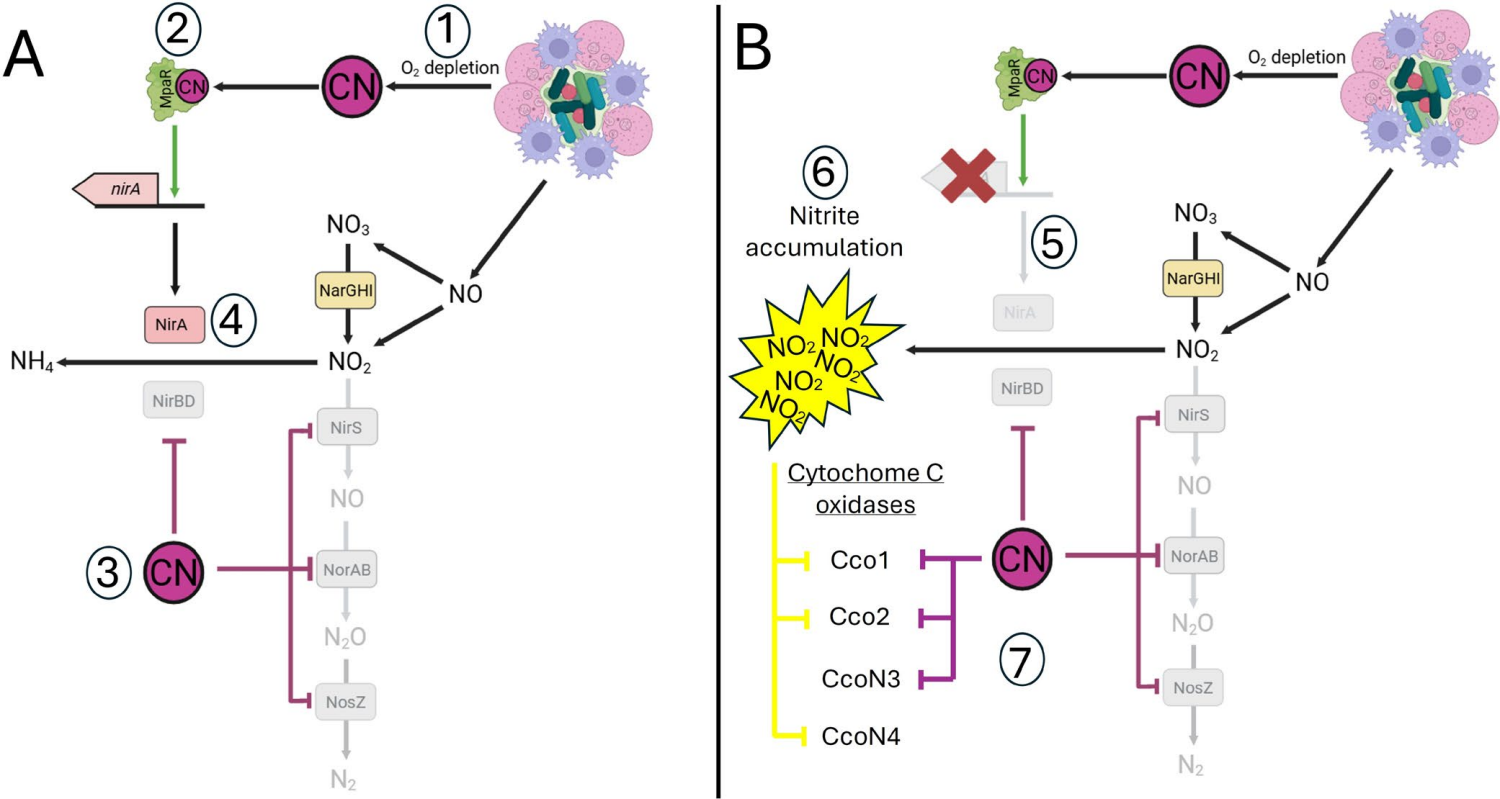
Proposed model for function of NirA in relation to other *P. aeruginosa* nitrite reductases NirB and NirS. (A1) Activity of PMN's and macrophages utilises locally dissolved oxygen and produces nitric oxide. Nitric oxide is oxidised to nitrite/nitrate whilst simultaneous O_2_ depletion triggers *P. aeruginosa* to produce HCN. (A2) Cyanide binds to MpaR activating transcription of NirA. (A3) Cyanide inhibits the heme containing enzymes of the denitrification pathway (NirS, NorAB and NosZ) and NirBD. Transcription of *nirBD* is only induced under N source limitation, in vivo amino acids are readily available inhibiting activation of *nirBD*. (A4) NirA reduces accumulating nitrite to ammonium. This ensures *P. aeruginosa* is not attempting to overcome multiple respiratory inhibitors simultaneously (CN and NO_2_). (B5) Model in absence of NirA through genetic manipulation or inhibition. (B6) In presence of cyanide and absence of NirA, no reductase is available to reduce nitrite to ammonium leading to accumulation. (B7) Impact of both nitrite and cyanide on *cbb*_3_‐type cytochrome c oxidases used by *P. aeruginosa* for aerobic respiration under reduced oxygen tensions. Cyanide and nitrite inhibit the Cco1 and Cco2 respiratory chains. Cyanide inhibits the nitrite‐resistant CcoN3 orphan cytochrome c oxidase catalytic sub‐unit. Conversely nitrite inhibits the cyanide‐resistant CcoN4 orphan cytochrome c catalytic sub‐unit. This may account for a role in NirA protecting CcoN4 from nitrite inhibition.

This is the first description of a cyanide‐resistant nitrite reductase, with siroheme‐dependent enzymes usually inhibited by cyanide. Other members of the PA4129‐34 cyanide‐inducible gene cluster have been linked to *P. aeruginosa* cyanide resistance. Arai and colleagues demonstrated that CcoN4 (PA4133) is a cyanide‐resistant cytochrome C oxidase catalytic sub‐unit that supports aerobic respiration at low‐oxygen tensions in the presence of cyanide (Hirai et al. 2016), whilst Smiley et al. confirmed that MpaR binds cyanide and activates transcription of the PA4129‐34 gene cluster (Smiley et al. 2024). Why *nirA* is required in this cyanide‐resistance gene cluster has yet to be determined; however, both cyanide and nitrite are respiratory inhibitors that often co‐occur during infection (La Rosa et al. 2019; Line et al. 2014; Arai 2011; Nair et al. 2014). In response to the presence of bacterial components, inducible nitric oxide synthase is activated in host cells, producing nitric oxide as an antimicrobial. Nitric oxide has a short half‐life and is highly reactive, forming potent antimicrobials such as peroxynitrite; however, the majority is auto oxidised to nitrite or nitrate (Figure 6) (Kolpen et al. 2014; Line et al. 2014). Meanwhile, *P. aeruginosa‐derived* cyanide is induced by the infection microenvironment, with the presence and activities of the host innate immune system resulting in use of locally dissolved oxygen, allowing ANR‐mediated upregulation of the *hcnABC* operon (Figure 6) (Kolpen et al. 2010; Kolpen et al. 2014; Pessi and Haas 2000). As a result of both cyanide and nitrite accumulation, mechanisms to detoxify or circumvent the action of both respiratory inhibitors are required. The cyanide‐insensitive oxidase (CioAB) has a low oxygen affinity, meaning it is not operational at low oxygen tensions, leaving CcoN4 as the only operational cyanide‐resistant cytochrome c oxidase (Arai 2011; Hirai et al. 2016). NirB is only upregulated when nitrite is present as a single N source (Romeo et al. 2012). NirS is structurally similar to cytochrome c oxidase and utilises cytochrome c as an electron donor; thus, this nitrite reductase and associated electron donor are inhibited by the presence of cyanide (Sun et al. 2002) (Figure 6). As a result, NirA will be the only nitrite reductase operational under cyanogenic conditions (Figure 6A). Loss of *nirA* results in the accumulation of both nitrite and cyanide, leaving *P. aeruginosa* susceptible to dual inhibition of anaerobic and aerobic respiration under reduced oxygen tensions (Figure 6B). This allows *P. aeruginosa* to circumvent and detoxify both self‐produced (CN) and host‐produced (NO/NO_2_) respiratory inhibitors, evading self‐poisoning. In addition to work surrounding CcoN4, Arai and colleagues demonstrated that CcoN3 is a nitrite‐resistant orphan cytochrome c oxidase. Similar to CcoN4, CcoN3 forms heterogeneous respiratory complexes with CcoO1/P1 and CcoO2/P2, allowing aerobic respiration to proceed in the presence of nitrite (Hirai et al. 2016). However, these complexes are inhibited by cyanide, meaning CcoN3 cannot facilitate aerobic respiration in the presence of both nitrite and cyanide (Figure 6B). Whilst nitrite may inhibit CcoN4, the action of NirA could protect *P. aeruginosa* from this intoxication, allowing aerobic respiration to proceed in the presence of both respiratory inhibitors (Figure 6B).

Protection from nitrative stress and cyanide is key to *P. aeruginosa* persistence and survival in hypoxic environments. Targeting the mechanisms required for adaptation to these environments could be a promising therapeutic target, with multiple targets now identified in the PA4129‐34 cyanide‐inducible operon. Through targeting NirA, CcoN4 or MpaR, we could promote *P. aeruginosa* self‐poisoning and prevent adaptation to the reduced oxygen environment typically encountered by *P. aeruginosa* in biofilms and during infection. Work is now ongoing to identify inhibitors of these systems for future therapeutic development.

## 4 Experimental Procedures

### 4.1 Bacterial Strains, Plasmids and Primers

Bacterial strains, plasmids and primers used in this study are listed in Table S1. Antibiotics were added when required at concentrations of 20 μg mL^−1^ for gentamicin, 15 μg mL^−1^ for nalidixic acid and carbenicillin 100 μg mL^−1^. Tetracycline was added at a final concentration of 150 μg mL^−1^ for *P. aeruginosa* and 10 μg mL^−1^ for *E. coli* . For agar pads used in colony biofilm assays, agar was added at 1.5% (w/v).

### 4.2 DNA Manipulations and Mutant Construction

Genomic DNA isolation was performed using the Wizard Genomic DNA purification kit (Promega). Plasmid isolation was performed with the GenElute plasmid miniprep kit (Sigma). All other standard DNA manipulation techniques such as restriction digest, ligation and transformation were performed according to the manufacturer's instructions.

PAO1‐L ∆*nirA* and complemented strains were previously generated (Fenn et al. 2021). To construct an in‐frame deletion in *nirB*, two DNA fragments consisting of 744 bp upstream and 791 bp downstream of *nirB* were amplified with Phusion polymerase (Thermo Scientific) and fused by overlap extension PCR. The upstream 744 bp fragment was amplified with primer pair nirBF1/nirBR1, whilst the downstream 791 bp fragment was amplified with primer pair nirBF2/nirBR2. Primers nirBF1/nirBR2 were modified with EcoRI and HindIII restriction sites, respectively. Primers nirBR1/nirBF1 were engineered with 15 bp complementary overhangs to facilitate overlap PCR. Overlap PCR performed using both DNA fragments followed, by a secondary PCR in which primers nirBF1/nirBR2 were added to generate the *nirB* deletion fragment. The final fragment was cloned into pME3087 (Girlanda et al. 2001) using EcoRI/HindIII restriction sites resulting in suicide plasmid pMEnirB. To construct an in‐frame deletion in NirS, the same procedure was followed, with the upstream 513 bp amplified with nirSF1/nirSR1 and the downstream 509 bp amplified with nirSF2/nirSR2. Primers nirSF1/nirSR2 were modified with KpnI and HindIII, with the final overlap nirS deletion fragment inserted into pME3087 using these restriction sites to form pME*nirS*.

The ∆*nirB*, ∆*nirAB*, ∆*nirS* and ∆*nirAS* deletion mutants were constructed by allelic exchange using pME*nirB* and pME*nirS*. Briefly, pME*nirB* and pME*nirS* were mobilised into PAO1‐L and PAO1‐L ∆*nirA* using *E. coli* S17.1 *λpir* (Metcalf et al. 1994). Conjugants were selected for on LB agar supplemented with tetracycline and nalidixic acid. Strains were restreaked twice on LB, with no antibiotic and subjected to tetracycline sensitivity enrichment to select for double crossover events. Colonies were screened for loss of resistance to tetracycline, with allelic exchange confirmed with PCR and DNA sequencing.

### 4.3 Growth Media and Kinetic Assays

*P. aeruginosa* and *E. coli* were routinely cultured in lysogeny broth (LB) at 37°C with shaking (200 rpm). Ammonium, nitrite and nitrate were added to the media at 10 mM, potassium cyanide was added at 100 μM, unless otherwise stated. MOPS‐succinate minimal media was formulated according to (LaBauve and Wargo 2012). Modified artificial sputum media were made according to (Fenn et al. 2021; Kirchner et al. 2012). Growth curves were performed in 50 mL conical flasks containing 10 mL of indicated media with a starting inoculum of 1 × 10^6^. At each timepoint, 200 μL of culture was removed from the conical, inserted into a 96‐well plate and optical density (OD_600nm_) was measured using a plate reader (Tecan infinite 200) at desired timepoints. Microaerobic conditions were maintained in a Donn‐Whitely M35 workstation using a 4‐gas mixture of O_2_ (2%), CO_2_ (5%), H_2_ (2%) and N_2_ (91%).

### 4.4 Colony Biofilm and CFU Determination

Colony biofilms were established on polycarbonate discs (Isopore membrane filter [Sigma]) with a diameter of 13 mm and pore size 0.2 μM, as previously performed in our lab (Robertson et al. 2024). Briefly, polycarbonate discs were treated for 10 min on each side in a benchtop UV cabinet to sterilise before use. Six‐well plates had 5 mL of LB or M‐ASM solidified with 1.5% agar inserted into each well. Sterile forceps were used to apply the sterile polycarbonate disc to the media. Bacterial strains were diluted to 1 × 10^5^ and 10 μL of this suspension was applied to each membrane, correlating to an initial inoculum of 1 × 10^3^ per polycarbonate disc. Inoculated plates were incubated at 37°C for 72 h, with polycarbonate discs transferred to fresh agar pads every 24 h.

At endpoint, polycarbonate discs were inserted into bijoux tubes containing 3 mL of PBS. Biofilms were vortexed for 30 s. Bijouxs were then floated in a sonicating water bath and sonicated for 15 min at 37 kHz. Disaggregated biofilms were then pelleted into 1 mL of PBS, and 10‐fold serial dilutions were performed; 20 μL of each sample was plated in triplicate on *Pseudomonas* isolation agar, and CFUs were calculated.

### 4.5 Nitrogen Source and Cyanide Transfer Growth Assay

Nitrite reductase mutants were cultured overnight in MOPS‐succinate‐NH_4_ at 37°C with aeration. Strains were normalised to 1 × 10^9^ CFU mL^−1^ and used to inoculate pre‐warmed MOPS‐succinate‐NH_4_, NH_4_ + KCN, NO_2_ and NO_2_ + KCN at 1 × 10^8^ CFU mL^−1^. Strains were incubated at 37°C with aeration for 24 h with CFU recovery enumerated at 0, 3, 6 and 24 h post inoculation. Cyanide was added to cultures at 50 μM where ever indicated.

### 4.6 Protein Overexpression and Purification

The *cysI orf* was amplified from PAO1‐L genomic DNA using primer pair NTcysI‐F/NTcysI‐R modified with an N‐terminal hexahistidyl tag and EcoRI/KpnI restriction sites. The modified *cysI* fragment was cloned into pSK67 using EcoRI/KpnI restriction sites, producing plasmid pSK*cysI*‐N. Purification of NirA and CysI was done as previously reported with minor modifications. Instead of using *E. coli* NiCo21 (DE3) as an overexpression host, the recently constructed *E. coli* BL21 (DE3) *suf* ^++^ strain was used. *E. coli* BL21 (DE3) *suf* ^++^ has had the *suf* iron–sulphur cluster biogenesis pathway genetically restored (Corless et al. 2020). With NirA and CysI encoding an iron–sulphur cluster, this strain was used to enhance the solubility and activity of these enzymes. Plasmids pSK4130‐N and pSK*cysI*‐N were co‐transformed into *E. coli* BL21 (DE3) *suf* ^++^ alongside pCDF‐*cysG*, with this plasmid promoting increased siroheme synthesis.

Protein overexpression was performed in terrific broth (TB) (24 g L^−1^ yeast extract, 12 g/L tryptone, 4% glycerol, 0.017 M KH_2_PO_4_, 0.072 M K_2_HPO_4_), supplemented with 1 mM FeSO_4_.6H_2_0. Single colonies were selected and grown in LB broth for 16 h at 37°C. Cell density was then adjusted to an optical density (OD) of 0.05 into TB and further grown to an OD of ∼0.6 to 0.8 (600 nm) at 37°C. Cultures were then cooled to 20°C before the addition of 0.05 M ferric citrate and induction with 0.1 mM isopropyl‐*β*‐d‐thiogalactopyranoside. Following a further 18‐h growth, cell pellets were harvested via centrifugation, flash frozen in liquid N_2_ and stored at −80°C. Recombinant NirA and CysI were subsequently purified using immobilised metal ion affinity chromatography (IMAC) and size‐exclusion chromatography (SEC) as previously reported (Fenn et al. 2021).

### 4.7 Nitrite and Sulphite Reduction Assays

NirA and CysI activity was tested using the artificial electron donor methyl‐viologen. The assay was adapted from Schnell and colleagues and performed under anaerobic conditions (Fenn et al. 2021; Schnell et al. 2005). Assays started following the addition of the desired electron acceptor. Potassium nitrite and hydrogen sulphite were used as electron acceptors for NirA and CysI, respectively. MV oxidation was tracked spectrophotometrically (A_605nm_) at 10 s intervals using a plate reader (Tecan infinite 200) fitted with a gas control unit to remove oxygen. Potassium cyanide (KCN) was added to reactions at denoted concentrations; NirA and CysI were preincubated with the relevant KCN concentration for 5 min prior to the addition of the electron acceptor to allow CN binding to siroheme.

## Author Contributions

**Samuel Fenn:** conceptualisation (equal), formal analysis (lead), investigation (lead), methodology (lead), project administration (supporting), visualisation (lead), Writing – original draft preparation (lead) and Writing – review and editing (equal). **Carey Lambert:** methodology (supporting), Validation (supporting), Writing – review and editing (equal). **Dimitra Panagiotopulou:** methodology (supporting), Writing – review and editing (equal). **Ruth Massey:** funding acquisition (supporting), Supervision (supporting), Resources (equal) and Writing – review and editing (equal). **Miguel Cámara:** conceptualisation (equal), Funding acquisition (lead), Project administration (lead), Resources (lead), Supervision (lead) and Writing – review and editing (equal).

## Funding

This work was supported by the Biotechnology and Biological Sciences Research Council (BB/R012415/1), Innovate UK (BB/X002950/1), Wellcome Trust (108876/Z/15/Z, 212258/Z/18/Z), Science Foundation Ireland (21/FFP‐A/9704).

## Conflicts of Interest

The authors declare no conflicts of interest.

## Supporting information


**Table S1:** Bacterial strains, plasmids and oligonucleotides.
**Figure S1:** Nitrite reductase mutant aerobic growth in LB supplemented with ammonium (A), nitrite (B) and nitrate (C). Under aerobic conditions, deletion of nitrite reductases did not compromise growth of *P. aeruginosa* on LB supplemented with ammonium, nitrite or nitrate. Nitrite mildly inhibited growth of *P. aeruginosa* , extending the lag phase by 2 h however, all single and double mutants demonstrated wild‐type growth kinetics.
**Figure S3:** Nitrite reductase mutant microaerobic growth in LB (A) and ASM (B) supplemented with ammonium (I) or nitrite (II). Microaerobic culture in the presence of ammonium did not impact replication of *P. aeruginosa* nitrite reductase mutants (A‐I and B‐I). When nitrite was present, mutation of both *nirA* and *nirS* impacted growth kinetics in LB (A‐II) and ASM (B‐II) under microaerobic conditions. This phenotype was not as pronounced as seen on MOPS‐succinate‐nitrite. Double deletion of *nirAS* severely impeded the ability of *P. aeruginosa* to replicate in the presence of nitrite under microaerobic conditions (A‐II and B‐II). This indicates that NirA and NirS work cooperatively to detoxify nitrite under reduced oxygenations.
**Figure S4:** Growth kinetics of *P. aeruginosa* nitrite reductase mutants when incubated aerobically in LB with KCN supplemented with ammonium (A) and nitrite (B). Addition of KCN extended the lag‐phase of *P. aeruginosa* whilst mechanisms of resistance were activated. Presence of both nitrite and cyanide resulted in retardation of ∆*nirA* mutant growth with complementation restoring the growth defect (B). Previously no phenotype was seen in the absence of cyanide under aerobic conditions (Figure S1B). No growth defect was exhibited by cultures supplemented with ammonium in the presence of cyanide indicating the function of *nirA* is specific to the presence of both nitrite and cyanide. The NirA‐dependent phenotype was similar to what is seen in Figure 3. The main difference is that a *nirB* mutant strain grew in LB due to availability of alternative nitrogen sources for growth, whilst in MOPS‐succinate‐nitrite the only nitrogen source is nitrite.
**Figure S5:** NirA functionally substitutes for NirB at high‐cell density in the presence of cyanide. PAO1‐L nitrite reductase mutant cultured overnight in MOPS‐succinate‐NH_4_ sub‐cultured at high cell density (1 × 10^8^) into MOPS‐succinate‐NH_4_ (A), NH_4_ + KCN (B), NO_2_ (C) and NO_2_ + KCN (D). Cyanide supplemented at 50 μM where applicable. Mutation of nitrite reductase mutants did not impair PAO1‐L use of ammonium as a single nitrogen source in the absence/presence of cyanide (A, B). When transferred to MOPS‐succinate‐nitrite, strains without a functional NirB failed to replicate and succumb to nitrite toxicity whilst loss of NirA demonstrates no significant effect (C). In comparison, when transferred to MOPS‐succinate‐NO_2_ + KCN a single *nirB* deletion mutant could replicate, whilst a *nirA* mutant was unable to grow (D). Loss of both *nirA* and *nirB* inhibited growth on nitrite+KCN indicating that NirA functionally substitutes for NirB at high cell density in the presence of cyanide. Mutation of *nirS* demonstrated no impact on these NirA‐ and NirB‐dependent phenotypes. Data displayed represent CFU recovery over a 24‐H period with static incubation at 37°C following transfer to the described condition. Data were collated from three independent experiments with at least three replicates. Points represent mean of experiments and error bars standard deviation.

## Data Availability

The authors confirm that the data supporting the findings of this study are available within the article and Supporting Information. Bacterial strains developed as part of this study are available upon request from the corresponding author S.F.

## References

[emi470256-bib-0001] Alford, M. A. , A. Baghela , A. T. Y. Yeung , D. Pletzer , and R. E. W. Hancock . 2020. “NtrBC Regulates Invasiveness and Virulence of *Pseudomonas Aeruginosa* During High‐Density Infection.” Frontiers in Microbiology 11: 773. 32431676 10.3389/fmicb.2020.00773 PMC7214821

[emi470256-bib-0002] Alford, M. A. , B. Baquir , A. An , K. Y. G. Choi , and R. E. W. Hancock . 2021. “NtrBC Selectively Regulates Host‐Pathogen Interactions, Virulence, and Ciprofloxacin Susceptibility of *Pseudomonas aeruginosa* .” Frontiers in Cellular and Infection Microbiology 11: 694789. 34249781 10.3389/fcimb.2021.694789 PMC8264665

[emi470256-bib-0003] Arai, H. 2011. “Regulation and Function of Versatile Aerobic and Anaerobic Respiratory Metabolism in *Pseudomonas Aeruginosa* .” Frontiers in Microbiology 2: 103. 21833336 10.3389/fmicb.2011.00103 PMC3153056

[emi470256-bib-0004] Boucher, H. W. , G. H. Talbot , J. S. Bradley , et al. 2009. “Bad Bugs, no Drugs: No ESKAPE! An Update From the Infectious Diseases Society of America.” Clinical Infectious Diseases: An Official Publication of the Infectious Diseases Society of America 48, no. 1: 1–12. 19035777 10.1086/595011

[emi470256-bib-0005] Broderick, K. E. , A. Chan , M. Balasubramanian , et al. 2008. “Cyanide Produced by Human Isolates of *Pseudomonas aeruginosa* Contributes to Lethality in *Drosophila melanogaster* .” Journal of Infectious Diseases 197, no. 3: 457–464. 18199034 10.1086/525282

[emi470256-bib-0006] Coleman, K. J. , A. Cornish‐Bowden , and J. A. Cole . 1978. “Purification and Properties of Nitrite Reductase From *Escherichia coli* K12.” Biochemical Journal 175, no. 2: 483–493. 217342 10.1042/bj1750483 PMC1186095

[emi470256-bib-0007] Corless, E. I. , E. L. Mettert , P. J. Kiley , and E. Antony . 2020. “Elevated Expression of a Functional Suf Pathway in *Escherichia Coli* BL21(DE3) Enhances Recombinant Production of an Iron‐Sulfur Cluster‐Containing Protein.” Journal of Bacteriology 202, no. 3: e00496‐19. 31712282 10.1128/JB.00496-19 PMC6964742

[emi470256-bib-0008] Fenn, S. , J. F. Dubern , C. Cigana , et al. 2021. “NirA Is an Alternative Nitrite Reductase From Pseudomonas Aeruginosa With Potential as an Antivirulence Target.” MBio 12, no. 2: e00207‐21. 33879591 10.1128/mBio.00207-21 PMC8092218

[emi470256-bib-0009] Frangipani, E. , I. Pérez‐Martínez , H. D. Williams , G. Cherbuin , and D. Haas . 2014. “A Novel Cyanide‐Inducible Gene Cluster Helps Protect *Pseudomonas aeruginosa* From Cyanide.” Environmental Microbiology Reports 6, no. 1: 28–34. 24596260 10.1111/1758-2229.12105

[emi470256-bib-0010] Freschi, L. , J. Jeukens , I. Kukavica‐Ibrulj , et al. 2015. “Clinical Utilization of Genomics Data Produced by the International *Pseudomonas aeruginosa* Consortium.” Frontiers in Microbiology 6: 1036. 26483767 10.3389/fmicb.2015.01036 PMC4586430

[emi470256-bib-0011] Gellatly, S. L. , and R. E. W. Hancock . 2013. “ *Pseudomonas aeruginosa* : New Insights Into Pathogenesis and Host Defenses.” Pathogens and Disease 67, no. 3: 159–173. 23620179 10.1111/2049-632X.12033

[emi470256-bib-0012] Girlanda, M. , S. Perotto , Y. Moenne‐Loccoz , et al. 2001. “Impact of Biocontrol *Pseudomonas Fluorescens* CHA0 and a Genetically Modified Derivative on the Diversity of Culturable Fungi in the Cucumber Rhizosphere.” Applied and Environmental Microbiology 67, no. 4: 1851–1864. 11282643 10.1128/AEM.67.4.1851-1864.2001 PMC92807

[emi470256-bib-0013] Hirai, T. , T. Osamura , M. Ishii , and H. Arai . 2016. “Expression of Multiple cbb3 Cytochrome c Oxidase Isoforms by Combinations of Multiple Isosubunits in *Pseudomonas Aeruginosa* .” Proceedings of the National Academy of Sciences of the United States of America 113, no. 45: 12815–12819. 27791152 10.1073/pnas.1613308113 PMC5111723

[emi470256-bib-0014] Kang, L. , J. LeGall , A. T. Kowal , and M. K. Johnson . 1987. “Spectroscopic Properties of Siroheme Extracted From Sulfite Reductases.” Journal of Inorganic Biochemistry 30, no. 4: 273–290. 3668524 10.1016/0162-0134(87)80071-5

[emi470256-bib-0015] Kaufman, J. , L. M. Siegel , and L. D. Spicer . 1993. “Proton NMR of *Escherichia coli* Sulfite Reductase: Studies of the Heme Protein Subunit With Added Ligands.” Biochemistry 32, no. 34: 8782–8791. 8395881 10.1021/bi00085a008

[emi470256-bib-0016] Khlebodarova, T. M. , N. A. Ree , and V. A. Likhoshvai . 2016. “On the Control Mechanisms of the Nitrite Level in *Escherichia Coli* Cells: The Mathematical Model.” BMC Microbiology 16, no. Suppl 1: 7. 26823079 10.1186/s12866-015-0619-x PMC4895483

[emi470256-bib-0017] Kirchner, S. , J. L. Fothergill , E. A. Wright , C. E. James , E. Mowat , and C. Winstanley . 2012. “Use of Artificial Sputum Medium to Test Antibiotic Efficacy Against *Pseudomonas Aeruginosa* in Conditions More Relevant to the Cystic Fibrosis Lung.” Journal of Visualized Experiments 64: e3857. 10.3791/3857 PMC3471314 22711026

[emi470256-bib-0018] Kolpen, M. , T. Bjarnsholt , C. Moser , et al. 2014. “Nitric Oxide Production by Polymorphonuclear Leucocytes in Infected Cystic Fibrosis Sputum Consumes Oxygen.” Clinical and Experimental Immunology 177, no. 1: 310–319. 24611476 10.1111/cei.12318 PMC4089181

[emi470256-bib-0019] Kolpen, M. , C. R. Hansen , T. Bjarnsholt , et al. 2010. “Polymorphonuclear Leucocytes Consume Oxygen in Sputum From Chronic *Pseudomonas aeruginosa* Pneumonia in Cystic Fibrosis.” Thorax 65, no. 1: 57–62. 19846469 10.1136/thx.2009.114512

[emi470256-bib-0020] La Rosa, R. , H. K. Johansen , and S. Molin . 2019. “Adapting to the Airways: Metabolic Requirements of *Pseudomonas Aeruginosa* During the Infection of Cystic Fibrosis Patients.” Metabolites 9, no. 10: 234. 31623245 10.3390/metabo9100234 PMC6835255

[emi470256-bib-0021] LaBauve, A. E. , and M. J. Wargo . 2012. “Growth and Laboratory Maintenance of *Pseudomonas Aeruginosa* .” Current Protocols in Microbiology 6: Unit 6E.1. 10.1002/9780471729259.mc06e01s25 PMC4296558 22549165

[emi470256-bib-0022] Létoffé, S. , Y. Wu , S. E. Darch , et al. 2022. “ *Pseudomonas Aeruginosa* Production of Hydrogen Cyanide Leads to Airborne Control of *Staphylococcus Aureus* Growth in Biofilm and in Vivo Lung Environments.” MBio 13, no. 5: e0215422. 36129311 10.1128/mbio.02154-22 PMC9600780

[emi470256-bib-0023] Li, W. , and C. D. Lu . 2007. “Regulation of Carbon and Nitrogen Utilization by CbrAB and NtrBC Two‐Component Systems in *Pseudomonas aeruginosa* .” Journal of Bacteriology 189, no. 15: 5413–5420. 17545289 10.1128/JB.00432-07 PMC1951800

[emi470256-bib-0024] Line, L. , M. Alhede , M. Kolpen , et al. 2014. “Physiological Levels of Nitrate Support Anoxic Growth by Denitrification of *Pseudomonas Aeruginosa* at Growth Rates Reported in Cystic Fibrosis Lungs and Sputum.” Frontiers in Microbiology 5: 554. 25386171 10.3389/fmicb.2014.00554 PMC4208399

[emi470256-bib-0025] Metcalf, W. W. , W. Jiang , and B. L. Wanner . 1994. “Use of the Rep Technique for Allele Replacement to Construct New *Escherichia coli* Hosts for Maintenance of R6K Gamma Origin Plasmids at Different Copy Numbers.” Gene 138, no. 1–2: 1–7. 8125283 10.1016/0378-1119(94)90776-5

[emi470256-bib-0026] Miller, W. R. , and C. A. Arias . 2024. “ESKAPE Pathogens: Antimicrobial Resistance, Epidemiology, Clinical Impact and Therapeutics.” Nature Reviews. Microbiology 22, no. 10: 598–616. 38831030 10.1038/s41579-024-01054-w PMC13147291

[emi470256-bib-0027] Nair, C. , A. Shoemark , M. Chan , et al. 2014. “Cyanide Levels Found in Infected Cystic Fibrosis Sputum Inhibit Airway Ciliary Function.” European Respiratory Journal 44, no. 5: 1253–1261. 25186256 10.1183/09031936.00097014

[emi470256-bib-0028] Pessi, G. , and D. Haas . 2000. “Transcriptional Control of the Hydrogen Cyanide Biosynthetic Genes hcnABC by the Anaerobic Regulator ANR and the Quorum‐Sensing Regulators LasR and RhlR in *Pseudomonas Aeruginosa* .” Journal of Bacteriology 182, no. 24: 6940–6949. 11092854 10.1128/jb.182.24.6940-6949.2000 PMC94819

[emi470256-bib-0029] Robertson, S. N. , M. Romero , S. Fenn , P. L. Kohler Riedi , and M. Cámara . 2024. “Development, Characterization, and Evaluation of a Simple Polymicrobial Colony Biofilm Model for Testing of Antimicrobial Wound Dressings.” Journal of Applied Microbiology 135, no. 3: lxae042. 38366933 10.1093/jambio/lxae042

[emi470256-bib-0030] Romeo, A. , E. Sonnleitner , T. Sorger‐Domenigg , M. Nakano , B. Eisenhaber , and U. Bläsi . 2012. “Transcriptional Regulation of Nitrate Assimilation in *Pseudomonas Aeruginosa* Occurs via Transcriptional Antitermination Within the nirBD‐PA1779‐cobA Operon.” Microbiology (Reading, England) 158, no. Pt 6: 1543–1552. 22493305 10.1099/mic.0.053850-0

[emi470256-bib-0031] Schnell, R. , T. Sandalova , U. Hellman , Y. Lindqvist , and G. Schneider . 2005. “Siroheme‐ and [Fe4‐S4]‐Dependent NirA From *Mycobacterium tuberculosis* Is a Sulfite Reductase With a Covalent Cys‐Tyr Bond in the Active Site.” Journal of Biological Chemistry 280, no. 29: 27319–27328. 15917234 10.1074/jbc.M502560200

[emi470256-bib-0032] Scoffield, J. A. , and H. Wu . 2016. “Nitrite Reductase Is Critical for *Pseudomonas Aeruginosa* Survival During Co‐Infection With the Oral Commensal *Streptococcus Parasanguinis* .” Microbiology 162, no. 2: 376–383. 26673783 10.1099/mic.0.000226 PMC4766596

[emi470256-bib-0033] Smiley, M. K. , D. C. Sekaran , F. Forouhar , et al. 2024. “MpaR‐Driven Expression of an Orphan Terminal Oxidase Subunit Supports *Pseudomonas Aeruginosa* Biofilm Respiration and Development During Cyanogenesis.” MBio 15, no. 1: e0292623. 38112469 10.1128/mbio.02926-23 PMC10790758

[emi470256-bib-0034] Sparacino‐Watkins, C. , J. F. Stolz , and P. Basu . 2014. “Nitrate and Periplasmic Nitrate Reductases.” Chemical Society Reviews 43, no. 2: 676–706. 24141308 10.1039/c3cs60249d PMC4080430

[emi470256-bib-0035] Sugishima, M. , K. Oda , T. Ogura , H. Sakamoto , M. Noguchi , and K. Fukuyama . 2007. “Alternative Cyanide‐Binding Modes to the Haem Iron in Haem Oxygenase.” Acta Crystallographica. Section F, Structural Biology Communications 63, no. Pt 6: 471–474. 10.1107/S174430910702475X PMC2335079 17554165

[emi470256-bib-0036] Sun, W. , M. Arese , M. Brunori , et al. 2002. “Cyanide Binding to Cd(1) Nitrite Reductase From *Pseudomonas Aeruginosa*: Role of the Active‐Site His369 in Ligand Stabilization.” Biochemical and Biophysical Research Communications 291, no. 1: 1–7. 11829453 10.1006/bbrc.2002.6391

[emi470256-bib-0037] Tiso, M. , and A. N. Schechter . 2015. “Nitrate Reduction to Nitrite, Nitric Oxide and Ammonia by Gut Bacteria Under Physiological Conditions.” PLoS One 10, no. 3: e0119712. 25803049 10.1371/journal.pone.0119712 PMC4372352

[emi470256-bib-0038] Turner, K. H. , J. Everett , U. Trivedi , K. P. Rumbaugh , and M. Whiteley . 2014. “Requirements for *Pseudomonas aeruginosa* Acute Burn and Chronic Surgical Wound Infection.” PLoS Genetics 10, no. 7: e1004518. 25057820 10.1371/journal.pgen.1004518 PMC4109851

[emi470256-bib-0039] Van Alst, N. E. , K. F. Picardo , B. H. Iglewski , and C. G. Haidaris . 2007. “Nitrate Sensing and Metabolism Modulate Motility, Biofilm Formation, and Virulence in *Pseudomonas aeruginosa* .” Infection and Immunity 75, no. 8: 3780–3790. 17526746 10.1128/IAI.00201-07 PMC1952006

[emi470256-bib-0040] Van Alst, N. E. , M. Wellington , V. L. Clark , C. G. Haidaris , and B. H. Iglewski . 2009. “Nitrite Reductase NirS Is Required for Type III Secretion System Expression and Virulence in the Human Monocyte Cell Line THP‐1 by *Pseudomonas aeruginosa* .” Infection and Immunity 77, no. 10: 4446–4454. 19651860 10.1128/IAI.00822-09 PMC2747934

[emi470256-bib-0041] Yeung, A. T. Y. , L. Janot , O. M. Pena , et al. 2014. “Requirement of the *Pseudomonas aeruginosa* CbrA Sensor Kinase for Full Virulence in a Murine Acute Lung Infection Model.” Infection and Immunity 82, no. 3: 1256–1267. 24379284 10.1128/IAI.01527-13 PMC3957987

