# Supplementary material for: NirA is a Cyanide‐Tolerant Nitrite Reductase Which Protects Pseudomonas aeruginosa From Self‐Poisoning
Source: Environ Microbiol Rep. 2025 Dec 9;17(6):e70256. doi: 10.1111/1758-2229.70256 (PMC12688703; doi:10.1111/1758-2229.70256)
Supplement: Supplementary file 1 — Table S1: Bacterial strains, plasmids and oligonucleotides. Figure S1: Nitrite reductase mutant aerobic growth in LB supplemented with ammonium (A), nitrite (B) and nitrate (C). Under aerobic conditions, deletion of nitrite reductases did not compromise growth of P. aeruginosa on LB supplemented with ammonium, nitrite or nitrate. Nitrite mildly inhibited growth of P. aeruginosa , extending the lag phase by 2 h however, all single and double mutants demonstrated wild‐type growth kinetics. Figure S3: Nitrite reductase mutant microaerobic growth in LB (A) and ASM (B) supplemented with ammonium (I) or nitrite (II). Microaerobic culture in the presence of ammonium did not impact replication of P. aeruginosa nitrite reductase mutants (A‐I and B‐I). When nitrite was present, mutation of both nirA and nirS impacted growth kinetics in LB (A‐II) and ASM (B‐II) under microaerobic conditions. This phenotype was not as pronounced as seen on MOPS‐succinate‐nitrite. Double deletion of nirAS severely impeded the ability of P. aeruginosa to replicate in the presence of nitrite under microaerobic conditions (A‐II and B‐II). This indicates that NirA and NirS work cooperatively to detoxify nitrite under reduced oxygenations. Figure S4: Growth kinetics of P. aeruginosa nitrite reductase mutants when incubated aerobically in LB with KCN supplemented with ammonium (A) and nitrite (B). Addition of KCN extended the lag‐phase of P. aeruginosa whilst mechanisms of resistance were activated. Presence of both nitrite and cyanide resulted in retardation of ∆nirA mutant growth with complementation restoring the growth defect (B). Previously no phenotype was seen in the absence of cyanide under aerobic conditions (Figure S1B). No growth defect was exhibited by cultures supplemented with ammonium in the presence of cyanide indicating the function of nirA is specific to the presence of both nitrite and cyanide. The NirA‐dependent phenotype was similar to what is seen in Figure 3. The main difference [file EMI4-17-e70256-s001.docx]

**Table S1 – Bacterial strains, plasmids and oligonucleotides**

| **Strains** | **Description** | **Origin** |
| --- | --- | --- |
| ***P. aeruginosa* strains** |  |  |
|  |  |  |
| PAO1-L | PAO1 Lausanne collection wild type |  |
| PAO1-L Δ*nirA* | In-frame marker-less deletion of *nirA* (PA4130) | (1) |
| PAO1-L Δ*nirA* pCTX*nirA* | In-frame marker-less deletion of *nirA* (PA4130) chromosomally complemented with *nirA* under the control of native promoter. | (1) |
| PAO1-L Δ*nirB* | In-frame marker-less deletion of *nirB* | This study |
| PAO1-L Δ*nirA* Δ*nirB* | Double deletion mutant of *nirA* and *nirB*. | This study |
| PAO1-L Δ*nirS* | In-frame marker-less deletion of *nirS* | This study |
| PAO1-L Δ*nirA* Δ*nirS* | Double deletion mutant of *nirA* and *nirS.* | This study |
| PA7 Bo599 | Clinical PA7 strain. | (2) |
| PA7 Bo599 Δ*nirA* | In-frame marker-less deletion of PA4130 orthologue. | (1) |
| PA14 AUS471 | Clinical PA14 strain. | (2) |
| PA14 AUS471 Δ*nirA* | In-frame marker-less deletion of PA4130 orthologue. | (1) |
| LESB58 PA-W39 | Clinical LESB58 strain isolated from wound. | (2) |
| LESB58 PA-W39 Δ*nirA* | In-frame marker-less deletion of PA4130 orthologue. | (1) |
|  |  |  |
| ***E. coli* strains** |  |  |
|  |  |  |
| NEB5-alpha | F−,φ80dl*acZ*ΔM15,Δ(*lacZYA-argF*)U169,*deoR*,*recA1*, *endA1*, *hsdR17*(rk−,mk+), *phoA*, *supE44*, λ−, thi1, *gyrA96*, *relA1.* | New England Biolabs |
| S17.1 λpir | pro, res^−^ *hsdR17* (rK^−^ mK^+^) *recA^−^* with an integrated RP4-2-Tc::Mu-Km::Tn7, Tp^r^ *λpir* | (3) |
| BL21(DE3)Suf^++^ | zdh-3632::cat, −26ATA−24 bp relative to *sufA* TSS changed to −26TAT−24 | (4) |
| **Plasmids** |  |  |
|  |  |  |
| pME3087 | Suicide vector, ColE1 replicon, Tc^R^ | (5) |
| pMEnirB | pME3087 based vector with upstream and downstream regions of *nirB* spliced together for marker-less deletion mutant generation. | This study |
| pMEnirS | pME3087 based vector with upstream and downstream regions of *nirS* spliced together for marker-less deletion mutant generation. | This study |
| Mini-CTX-1 | *aatB* P. aeruginosa integrative vector, Tc | (6) |
| pCTXnirA | Integrative *nirA* complementation vector under control of the native promoter (+498bp), Tc^R^ | (1) |
| Mini-CTX-lux-Gm | aatB *P. aeruginosa* integrative vector containing *luxABCDE* cassette. | Lab strain |
| pLux-P*_nirA_* | Transcriptional reporter with *nirA* fused upstream of *luxABCDE* in mini-CTX-lux-Gm | This study |
| pLux-P*_nirB_* | Transcriptional reporter with *nirA* fused upstream of *luxABCDE* in mini-CTX-lux-Gm | This study |
| pSK67 | pTOPO type vector for protein overexpression. Under control of a T7 promoter and IPTG inducible. Amp^R^ | Lab strain |
| pSK4130-N | pSK67 based vector with N-terminal hexahistidyl tagged *nirA* inserted at the EcoRI and SacI restriction sites for overexpression | (1) |
| PSKcysI-N | pSK67 based vector with N-terminal hexahistidyl tagged *cysI* inserted at the EcoRI and SacI restriction sites for overexpression | This study |
| pCDF-DUET1 | DUET vector containing 2 T7 promoters under the control of LacI. Enables co-expression of up to 4 target proteins. Sp^R^ | Novagen |
| pCDF-*cysG* | pCDF-DUET1 based vectors with *cysG* inserted into MCS2 at the NcoI and XhoI site for overexpresssion | (1) |
|  |  |  |
| **Primers** | **Sequence** | **Modifications** |
| PnirA_F | 5’-ATAGGTACCGGCCGTTCACCGCCGACG-3’ | KpnI |
| PnirA_R | 5’-TATGGATCCCGCAAAGCCCTCATCGACAG-3’ | BamHI |
| PnirB_F | 5’-ATAGGTACCGCCTGGATCGACGACCTGCTG-3’ | KpnI |
| PnirB_R | 5’TATGGATCCGCGGAGTAGCTCCTGCATAAG-3’ | BamHI |
| NirBF1 | 5’-ATAGAATTCGCTGCGCCTGCTGGATTTCGG-3’ | EcoRI |
| NirBR1 | 5’-GCAAGGGATTCAGACGTTCTTCTTCATGCGGAGTAGCTCCTG-3’ | N/A |
| NirBF2 | 5’-CTACTCCGCATGAACAAGAACGTCTGAATCCCTTGCCCGGGC-3’ | N/A |
| NirBR2 | 5’-TATAAGCTTCGCCAGCTTGTTGAAGGCGTAGTAG-3’ | HindIII |
| NirSF1 | 5’-ATAGGTACCGCAGATACCGCCTTCGCGCAC-3’ | KpnI |
| NirSR1 | 5’-CGGGTCTCAGTACACGCCAAATGGCATGGTCTATCTCCTC-3’ | N/A |
| NirSF2 | 5’-ACCATGCCATTTGGCGTGTACTGAGACCCGCGTGCG-3’ | N/A |
| NirSR2 | 5’-ATAAAGCTTGCAGGTGACGCAACTGGGCCT-3’ | HindIII |
| NTCysI_F | 5’ATAGAATTCATGCATCACCATCACCATCACAGCGAAAAACATCCAGGGCC-3’ | EcoRI, 6XHis |
| NTCysI_R | 5’-TATGGTACCTTAATCCCACAAATCACGCGCC | KpnI |

**Figure S1 – Nitrite reductase mutant aerobic growth in LB supplemented with ammonium (A), nitrite (B) and nitrate (C).** Under aerobic conditions, deletion of nitrite reductases did not compromise growth of *P. aeruginosa* on LB supplemented with ammonium, nitrite or nitrate. Nitrite mildly inhibited growth of P. aeruginosa, extending the lag phase by 2 hours however, all single and double mutants demonstrated wild-type growth kinetics.


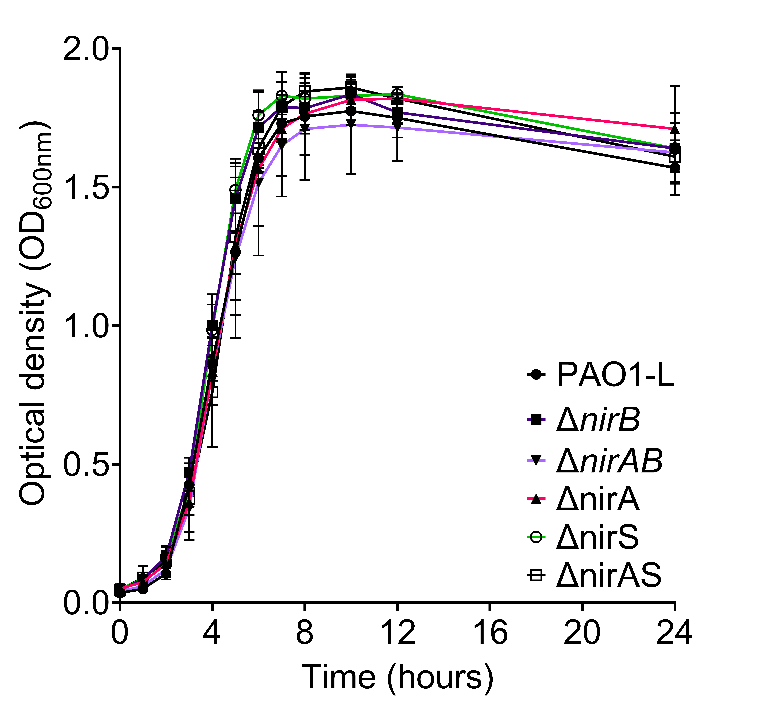


C

B

A


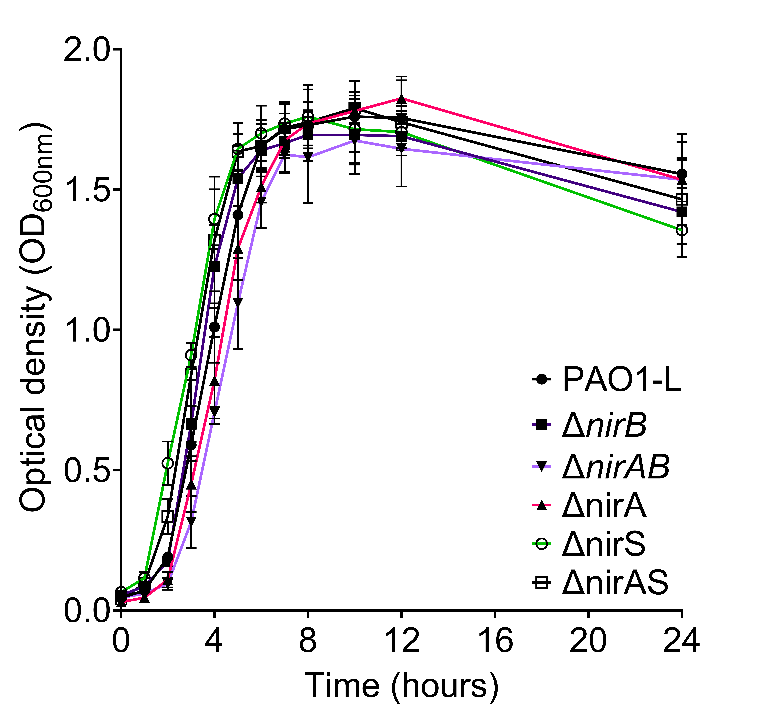

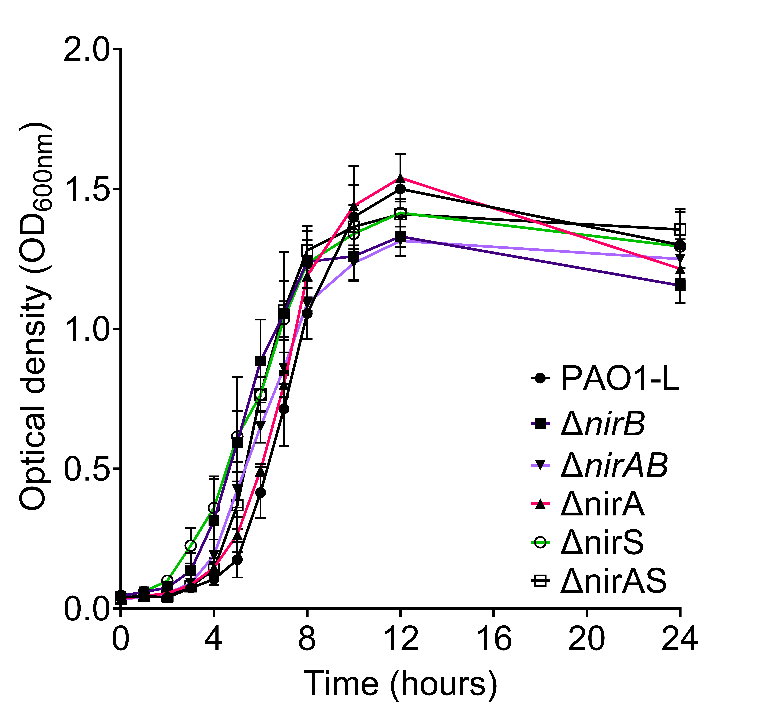


**Figure S2 – ASM colony biofilms supplemented with ammonium, nitrite and nitrate under aerobic (A) and microaerobic (B) conditions.** ASM media was chosen to replicate *in vivo* environment associated with reduced oxygenation. Deletion of *nirA* compromised CFU recovery from colony biofilms under aerobic conditions in the presence of nitrite and nitrate (A). This phenotype was exacerbated under reduced oxygenation with deletion of *nirA* resulting in a 1-log reduction and 2-log reduction in CFU recovery when cultured on nitrite and nitrate respectively (B). Restoration of *nirA* restored CFU recovery under normoxic (A) and microoxic (B) conditions.

BA

A


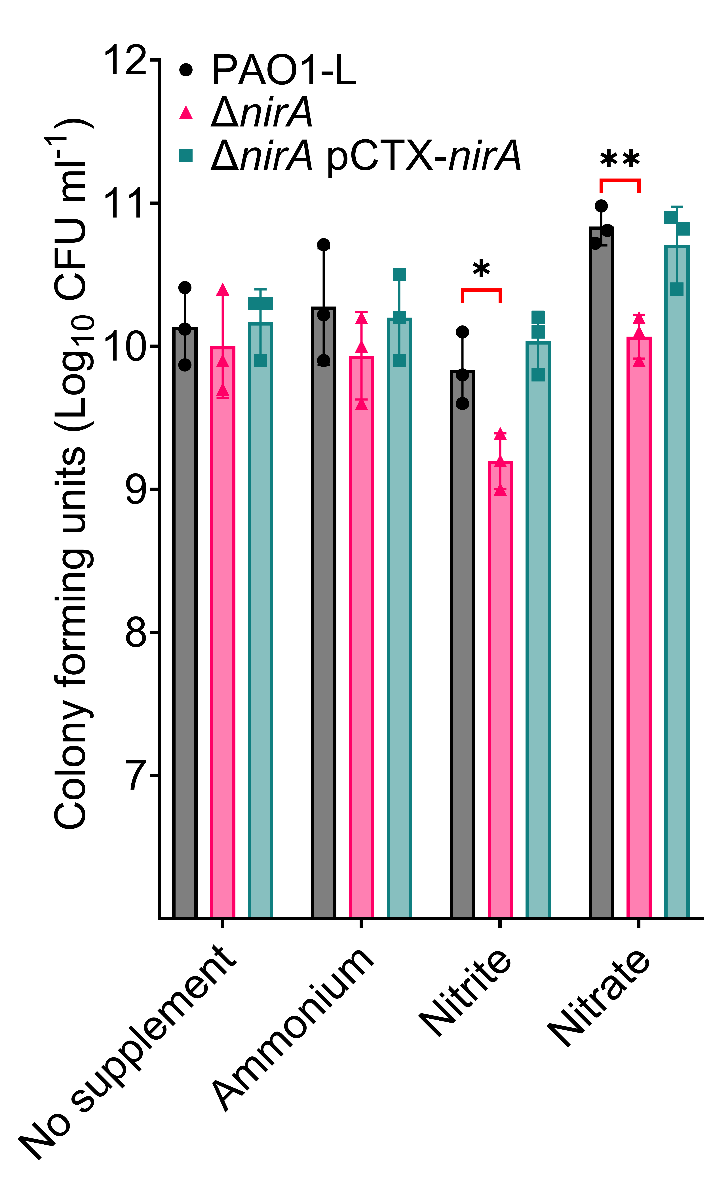

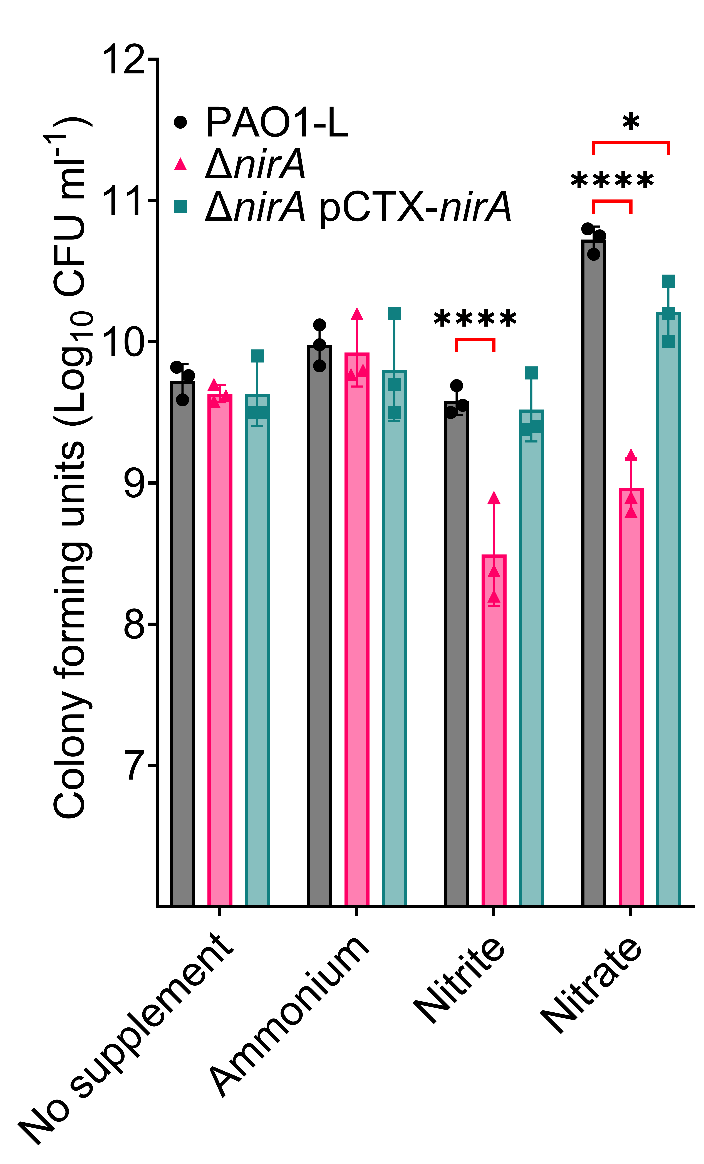


**Figure S3 – Nitrite reductase mutant microaerobic growth in LB (A) and ASM (B) supplemented with ammonium (I) or nitrite (II).** Microaerobic culture in the presence of ammonium did not impact replication of *P. aeruginosa* nitrite reductase mutants (A-I and B-I). When nitrite was present, mutation of both *nirA* and *nirS* impacted growth kinetics in LB (A-II) and ASM (B-II) under microaerobic conditions. This phenotype was not as pronounced as seen on MOPS-succinate-nitrite. Double deletion of *nirAS* severely impeded the ability of *P. aeruginosa* to replicate in the presence of nitrite under microaerobic conditions (A-II and B-II). This indicates that NirA and NirS work cooperatively to detoxify nitrite under reduced oxygenations.


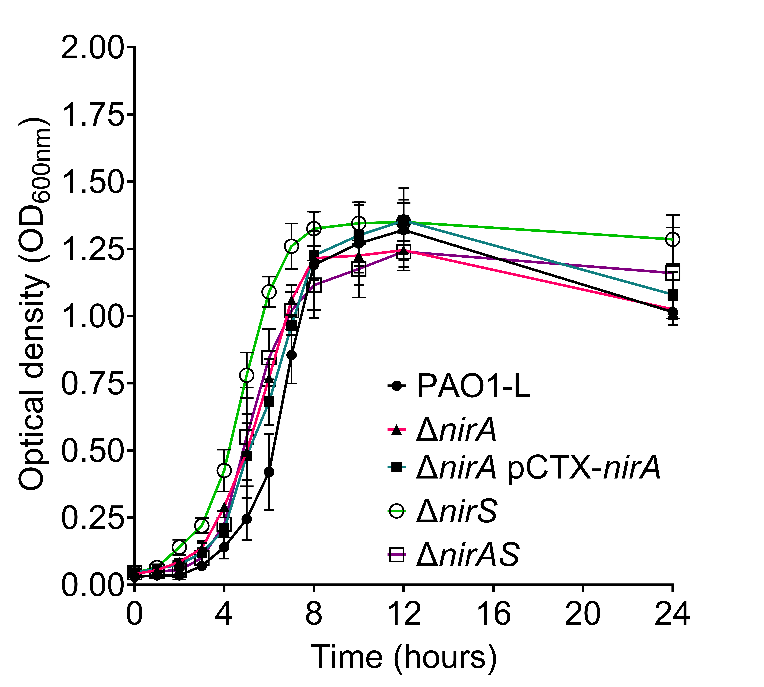

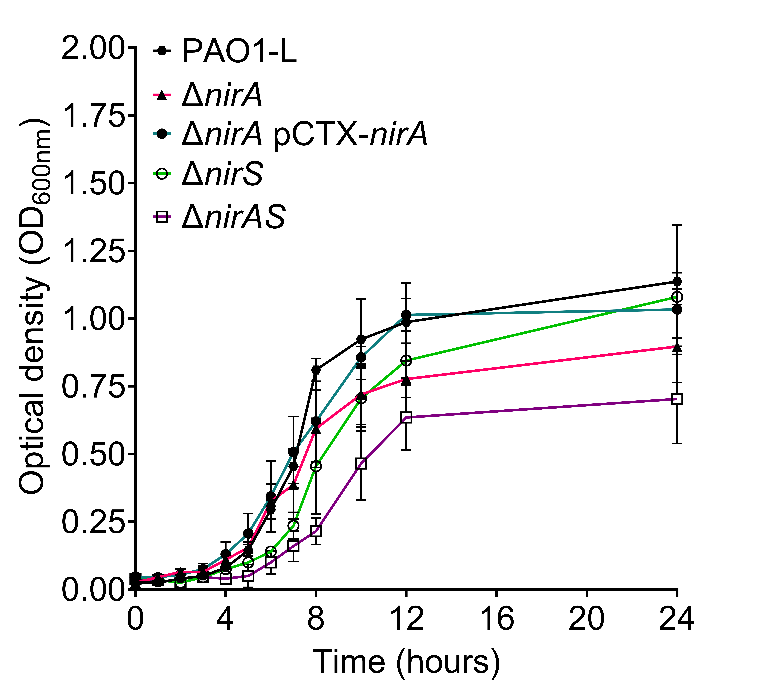

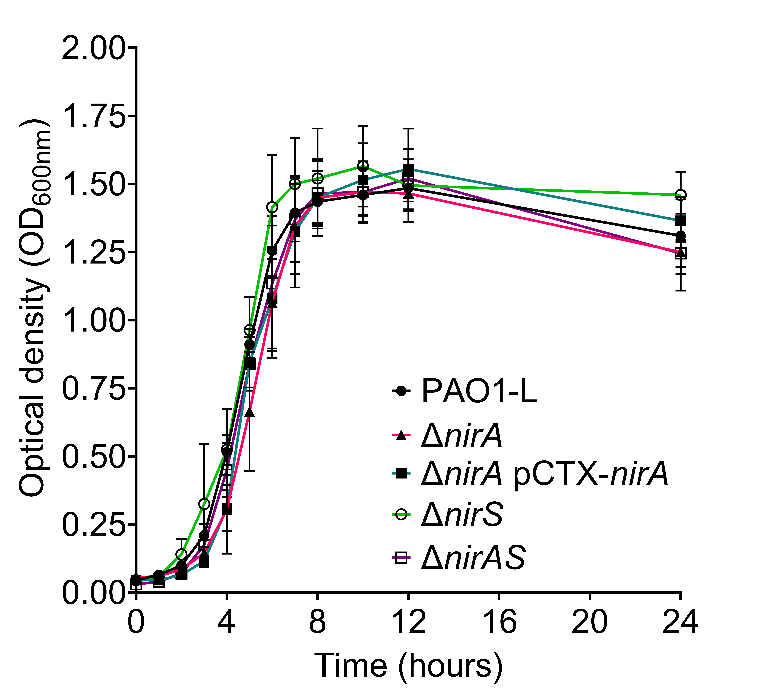

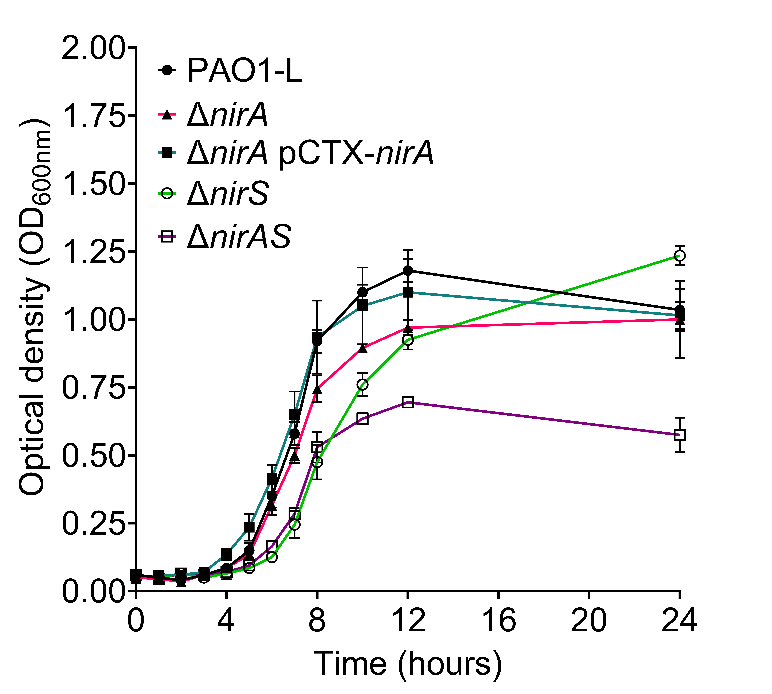


B-II

B-I

A-II

A-I

**Figure S4 – Growth kinetics of *P. aeruginosa* nitrite reductase mutants when incubated aerobically in LB with KCN supplemented with ammonium (A) and nitrite (B).** Addition of KCN extended the lag-phase of *P. aeruginosa* whilst mechanisms of resistance were activated. Presence of both nitrite and cyanide resulted in retardation of ∆*nirA* mutant growth with complementation restoring the growth defect (B). Previously no phenotype was seen in the absence of cyanide under aerobic conditions (Figure S1-B). No growth defect was exhibited by cultures supplemented with ammonium in the presence of cyanide indicating the function of *nirA* is specific to the presence of both nitrite and cyanide. The NirA-dependent phenotype was similar to what is seen in Figure 3. The main difference is that a *nirB* mutant strain grew in LB due to availability of alternative nitrogen sources for growth, whilst in MOPS-succinate-nitrite the only nitrogen source is nitrite.


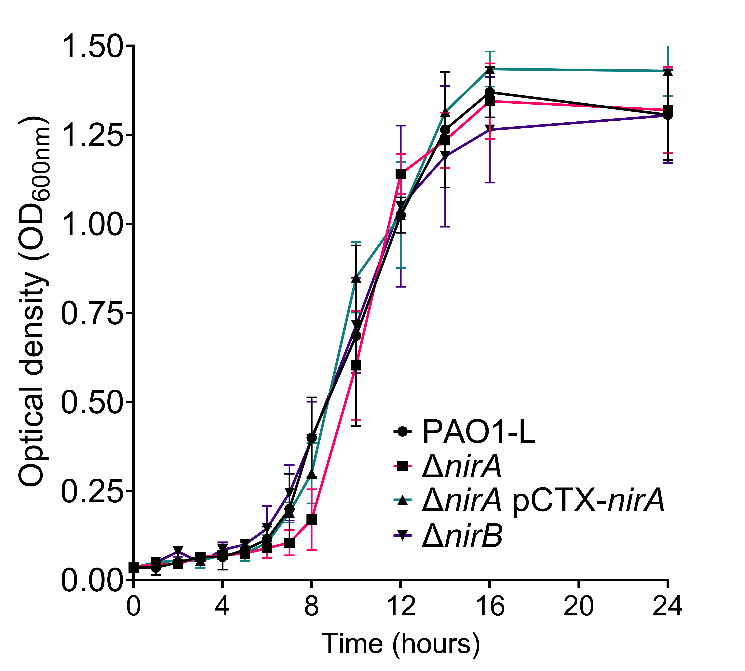

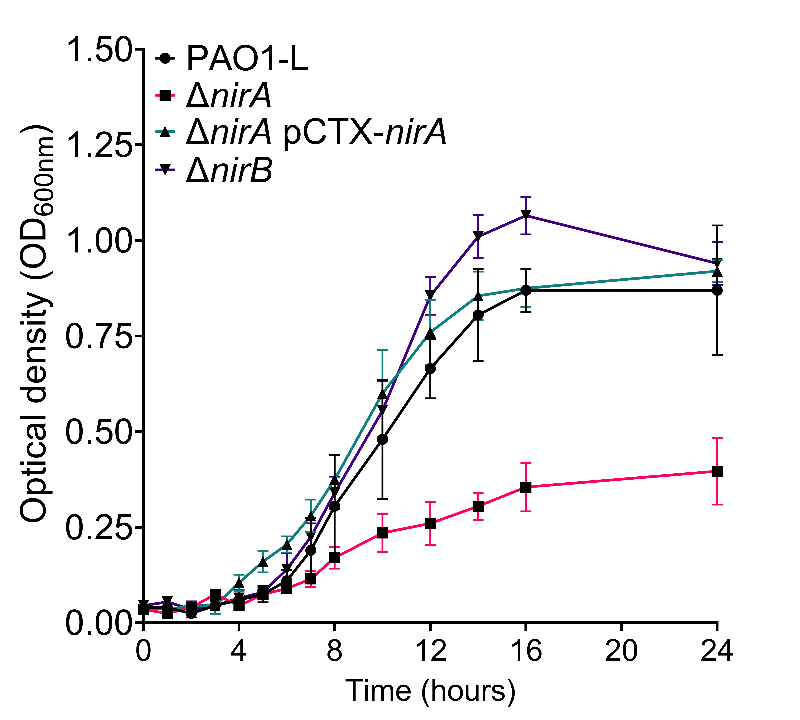


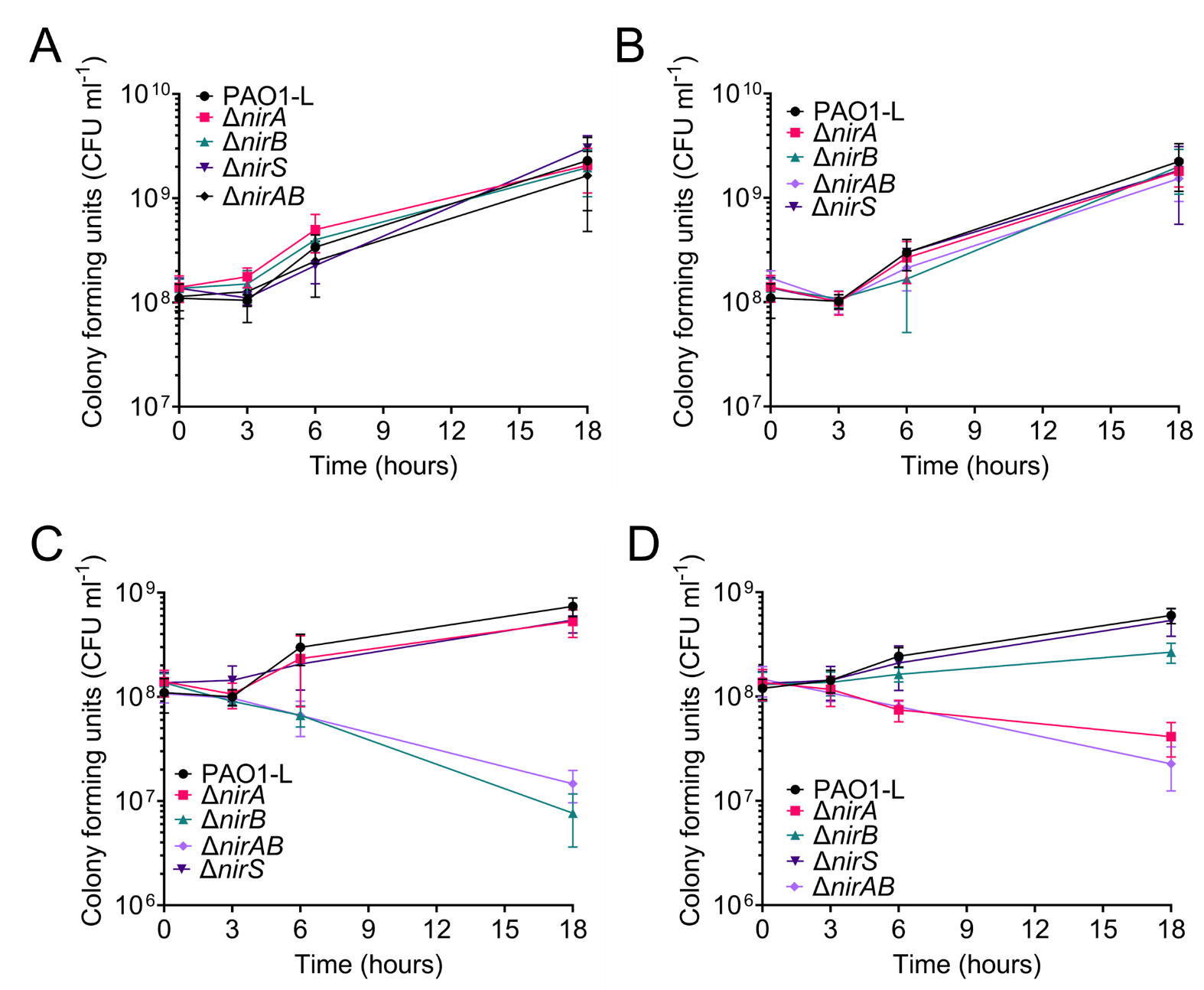
**Figure S5 – NirA functionally substitutes for NirB at high-cell density in the presence of cyanide.** PAO1-L nitrite reductase mutant cultured overnight in MOPS-succinate-NH_4_ sub-cultured at high cell density (1x10^8^) into MOPS-succinate-NH_4_ (**A**), NH_4_+KCN (**B**), NO_2_ (**C**), and NO_2_+KCN (**D**). Cyanide supplemented at 50 µM where applicable. Mutation of nitrite reductase mutants did not impair PAO1-L use of ammonium as a single nitrogen source in the absence/ presence of cyanide (**A-B**). When transferred to MOPS-succinate-nitrite, strains without a functional NirB failed to replicate and succumb to nitrite toxicity whilst loss of NirA demonstrates no significant effect (**C**). In comparison, when transferred to MOPS-succinate-NO_2_+KCN a single *nirB* deletion mutant could replicate, whilst a *nirA* mutant was unable to grow (**D**). Loss of both *nirA* and *nirB* inhibited growth on nitrite+KCN indicating that NirA functionally substitutes for NirB at high cell density in the presence of cyanide. Mutation of *nirS* demonstrated no impact on these NirA and NirB dependent phenotypes. Data displayed represents CFU recovery over a 24-H period with static incubation at 37^°^C following transfer to the described condition. Data collated from three independent experiments with at least three replicates. Points represent mean of experiments and error bars standard deviation.

**Supplementary references**

1. Fenn S, Dubern JF, Cigana C, De Simone M, Lazenby J, Juhas M, et al. NirA Is an Alternative Nitrite Reductase from *Pseudomonas aeruginosa* with Potential as an Antivirulence Target. mBio. 2021 Apr 20;12(2):e00207-21.

2. Freschi L, Jeukens J, Kukavica-Ibrulj I, Boyle B, Dupont MJ, Laroche J, et al. Clinical utilization of genomics data produced by the international *Pseudomonas aeruginosa* consortium. Front Microbiol. 2015;6:1036.

3. Metcalf WW, Jiang W, Wanner BL. Use of the rep technique for allele replacement to construct new *Escherichia coli* hosts for maintenance of R6K gamma origin plasmids at different copy numbers. Gene. 1994 Jan 28;138(1–2):1–7.

4. Corless EI, Mettert EL, Kiley PJ, Antony E. Elevated Expression of a Functional Suf Pathway in *Escherichia coli* BL21(DE3) Enhances Recombinant Production of an Iron-Sulfur Cluster-Containing Protein. J Bacteriol. 2020 Jan 15;202(3):e00496-19.

5. Girlanda M, Perotto S, Moenne-Loccoz Y, Bergero R, Lazzari A, Defago G, et al. Impact of biocontrol *Pseudomonas fluorescens* CHA0 and a genetically modified derivative on the diversity of culturable fungi in the cucumber rhizosphere. Appl Environ Microbiol. 2001 Apr;67(4):1851–64.

6. Hoang TT, Kutchma AJ, Becher A, Schweizer HP. Integration-proficient plasmids for *Pseudomonas aeruginosa*: site-specific integration and use for engineering of reporter and expression strains. Plasmid. 2000 Jan;43(1):59–72.
